# Bloch‐Siegert $B_{1}^{+}$‐mapping for human cardiac ^31^P‐MRS at 7 Tesla

**DOI:** 10.1002/mrm.26005

**Published:** 2015-10-28

**Authors:** William T. Clarke, Matthew D. Robson, Christopher T. Rodgers

**Affiliations:** ^1^ Oxford Centre for Clinical Magnetic Resonance Research (OCMR)University of OxfordLevel 0, John Radcliffe HospitalOxfordOX3 9DUUnited Kingdom

**Keywords:** ^31^P‐MRS, phosphorus, Bloch‐Siegert, $B_{1}^{+}$‐mapping, spectroscopy, cardiac, heart, 7T

## Abstract

**Purpose:**

Phosphorus MR spectroscopy (^31^P‐MRS) is a powerful tool for investigating tissue energetics in vivo. Cardiac ^31^P‐MRS is typically performed using surface coils that create an inhomogeneous excitation field across the myocardium. Accurate measurements of $B_{1}^{+}$ (and hence flip angle) are necessary for quantitative analysis of ^31^P‐MR spectra. We demonstrate a Bloch‐Siegert $B_{1}^{+}$‐mapping method for this purpose.

**Theory and Methods:**

We compare acquisition strategies for Bloch‐Siegert $B_{1}^{+}$‐mapping when there are several spectral peaks. We optimize a Bloch‐Siegert sensitizing (Fermi) pulse for cardiac ^31^P‐MRS at 7 Tesla (T) and apply it in a three‐dimensional (3D) chemical shift imaging sequence. We validate this in phantoms and skeletal muscle (against a dual‐TR method) and present the first cardiac ^31^P $B_{1}^{+}$‐maps at 7T.

**Results:**

The Bloch‐Siegert method correlates strongly (Pearson's r = 0.90 and 0.84) and has bias <25 Hz compared with a multi‐TR method in phantoms and dual‐TR method in muscle. Cardiac 3D $B_{1}^{+}$‐maps were measured in five normal volunteers. $B_{1}^{+}$ maps based on phosphocreatine and alpha‐adenosine‐triphosphate correlated strongly (r = 0.62), confirming that the method is T_1_ insensitive.

**Conclusion:**

The 3D ^31^P Bloch‐Siegert $B_{1}^{+}$‐mapping is consistent with reference methods in phantoms and skeletal muscle. It is the first method appropriate for ^31^P $B_{1}^{+}$‐mapping in the human heart at 7T. Magn Reson Med 76:1047–1058, 2016. © 2015 The Authors. Magnetic Resonance in Medicine published by Wiley Periodicals, Inc. on behalf of International Society for Magnetic Resonance in Medicine. This is an open access article under the terms of the Creative Commons Attribution License, which permits use, distribution and reproduction in any medium, provided the original work is properly cited.

## INTRODUCTION

Phosphorus magnetic resonance spectroscopy (^31^P‐MRS) provides unique insight into the metabolism of the human heart in vivo. ^31^P‐MRS studies have revealed the role of the creatine kinase energy shuttle—and its constituent “high energy phosphate” metabolites—in supplying energy to drive the contractile work of the heart 1, 2. ^31^P‐MRS has been used to study a range of cardiomyopathies and to stratify risk in patient groups at 1.5 Tesla (T), 3T, and now recently with improved signal‐to‐noise ratios (SNRs) at 7T 3, 4, 5.

In principal, MR spectra provide a quantitative measure of metabolite concentrations in vivo. Yet, because ^31^P‐MR spectra typically have $∼10^{-5}$ x lower $SNR/\sqrt{t}$ than ^1^H MRI, and ^31^P‐containing metabolites have T_1_ relaxation times of several seconds in vivo, it is not normally practical to obtain fully relaxed ^31^P‐MR spectra in vivo. Using a repetition time (TR) < 5 × T_1_ causes partial saturation. So it is essential to know the flip angle in each voxel (and the metabolite T_1_) if we are to determine accurate metabolite concentrations, or metabolite concentration ratios 6.

The phosphorus gyromagnetic ratio γP31=17.235 MHz T^‐1^_,_ is only 40% of γH1, so there is less nutation of the magnetization for a given radiofrequency (RF) transmit field strength $\left(B_{1}^{+}\right)$. To mitigate both the low SNR of cardiac ^31^P‐MRS and the detrimental effect of the low gyromagnetic ratio on the transmit performance surface coils are often used to maximize both $B_{1}^{+}$ and the peak receive sensitivity ( $B_{1}^{-}$). However, surface coils give large variations in $B_{1}^{+}$ depending on coil placement and the location of the voxel of interest in the heart.

$B_{1}^{+}$‐insensitive adiabatic excitation pulses have been used to obtain uniform excitation flip angles despite this $B_{1}^{+}$‐inhomogeneity at 1.5T and 3T, thereby enabling absolute quantification of metabolite concentrations 7, 8. But in the heart at 7T, the wide bandwidth of ^31^P spectra, the limited peak $B_{1}^{+}$ and the regulatory limits on specific absorption rate (SAR) make adiabatic excitation challenging.

Approaches that compute $B_{1}^{+}$ using the Biot‐Savart law or finite‐differences time‐domain simulations, or that measure $B_{1}^{+}$ in advance in a phantom are demanding, because dielectric effects make the spatial distribution of $B_{1}^{+}$ depend on each subject's anatomy 9—and more so at 7T than at 1.5T or 3T 10. Therefore, at 7T, because it is challenging to use $B_{1}^{+}$‐insensitive adiabatic pulses and because calculated field maps are increasingly inaccurate, it is essential to be able to directly measure, for each subject, the spatial distribution of $B_{1}^{+}$ in the human heart, which is the focus of this work.

There is a limited choice of existing $B_{1}^{+}$‐mapping methods that are compatible with cardiac ^31^P‐MRS at 7T. The dual‐TR, flip‐angle measurement method of Chmelik et al is effective in skeletal muscle, but it relies on knowledge of the metabolite T_1_ values 11. This is particularly limiting for the highest SNR peak, phosphocreatine (PCr), whose apparent T_1_ depends on the creatine‐kinase exchange rate 12, which is known to change markedly in heart failure 13.

A second potential method is actual flip‐angle imaging (AFI), which has been used for musculoskeletal ^31^P‐MRS at 7T 14. However, that protocol used a total TR of 4 s; a single average, 16 × 16 × 8 matrix, acquisition weighted, three‐dimensional (3D) chemical shift imaging (CSI) acquisition would take 40 min, leaving insufficient time for the main acquisition in a patient study.

The above methods illustrate some of the classic difficulties associated with ^31^P‐MRS and X‐nuclear MRS in general, where long, and potentially unknown T_1_ values, combined with low SNR, limit the ability to measure accurately a fine change in signal magnitude, hampering the translation of methods from ^1^H‐MRI and ^1^H‐MRS. We, therefore, introduce a novel method for $B_{1}^{+}$‐mapping in multivoxel spectroscopy based on the Bloch‐Siegert shift 15. This approach encodes the $B_{1}^{+}$ measurement in the phase of the magnetisation, so it is independent of the TR and of metabolite T_1_ values.

^31^P‐MRS, and other X‐nuclear systems, typically have complex multi‐peak spectra, therefore, we explore how a Bloch‐Siegert module can be applied for multi‐peak spectroscopy and derive expressions for the accuracy and precision of Bloch‐Siegert spectroscopy $B_{1}^{+}$‐mapping.

Finally, we optimize a protocol for cardiac ^31^P Bloch‐Siegert spectroscopy $B_{1}^{+}$‐mapping at 7T. We validate this protocol in phantoms, in human skeletal muscle and demonstrate it in the hearts of five normal volunteers at 7T.

## THEORY

### Bloch‐Siegert $B_{1}^{+}$ Mapping

Bloch‐Siegert $B_{1}^{+}$‐mapping uses an additional off‐resonance RF pulse (a “Bloch‐Siegert pulse”) inserted between excitation and readout in a pulse sequence. During this Bloch‐Siegert pulse, there is a transient Bloch‐Siegert shift in the Larmor precession frequency 16, 17. This causes an accumulated phase shift of the transverse magnetization and hence alters the phase of the spectral peak. The Bloch‐Siegert pulse is placed at a large frequency offset ( $\omega_{RF}\gg \gamma B_{1}^{+}$) from the measured peak, to minimize additional excitation of magnetization by the Bloch‐Siegert pulse (“direct excitation”), and to give a linear relationship between the Bloch‐Siegert phase shift and $B_{1}^{+}$ 18. In this linear regime, the additional phase $\phi_{BS}$ (in radians) accumulated by a species over the duration *T*_p_ of the Bloch‐Siegert pulse is

(1) $$\phi_{\text{BS}}=2\pi \int_{0}^{T_{p}}\frac{|\gamma B_{1}^{+}(t){|}^{2}}{2\omega_{\text{RF}}}dt=\frac{\pi \gamma^{2}B_{1,\text{normalized}}^{2}}{\omega_{\text{RF}}}\times {\left(B_{1,\text{peak}}^{+}\right)}^{2}$$

where *t* is time (in s), $\omega_{\text{RF}}$ is the frequency offset (in Hz) from the Bloch‐Siegert pulse to the metabolite of interest, γ is the gyromagnetic ratio (in Hz T^‐1^), $B_{1}^{+}(t)$ is the time dependent pulse amplitude of the Bloch‐Siegert pulse (in T), $B_{1,\text{peak}}^{+}$ is the maximum amplitude of the Bloch‐Siegert pulse (in T), and $B_{1,\text{normalized}}^{2}=\tilde{B}=\int_{0}^{T_{P}}B_{1}^{+}{\left(t\right)}^{2}dt/{\left(B_{1,\text{peak}}^{+}\right)}^{2}$ is the normalized pulse‐envelope squared‐integral of the Bloch‐Siegert pulse.

In the original ^1^H imaging Bloch‐Siegert $B_{1}^{+}$‐mapping method 15, two images are acquired with Fermi‐shaped 19 Bloch‐Siegert pulses at equal and opposite frequency offsets $\pm \omega_{\text{RF}}$ relative to the water resonance. In each voxel, the images have phase $\phi_{i}=\phi_{0}+\phi_{\text{BS}}$, where $\phi_{0}$ is the normal phase due to the coil transmit and receive phases, B_0_ inhomogeneity, sequence dead‐time, flow, etc.. $B_{1}^{+}$ is then obtained from the phase difference between the two images

(2) $$\phi_{2}-\phi_{1}=\left(\phi_{0}+\phi_{\text{BS}}{|}_{+\omega_{\text{RF}}}\right)-\left(\phi_{0}+\phi_{\text{BS}}{|}_{-\omega_{\text{RF}}}\right)=2\phi_{\text{BS}}.$$

Using Eq. (1), $\gamma B_{1,\text{peak}}^{+}$ is given by:

(3) $$\gamma B_{1,\text{peak}}^{+}=\sqrt{\frac{(\phi_{2}-\phi_{1})\omega_{\text{RF}}}{2\pi \tilde{B}}}.$$

### Possible Approaches When There Are Multiple Peaks

In ^31^P‐MRS, and X‐nuclear MRS in general, multiple resonances are observed and there is often no dominant peak (unlike ^1^H‐MR where the water peak dominates). Therefore, for a single choice of Bloch‐Siegert pulse center frequency, each peak in the spectrum will experience a different frequency offset, $\omega_{\text{RF}}$, from the Bloch‐Siegert pulse to that metabolite, and therefore, according to Eq. (1), a different Bloch‐Siegert phase shift.

As the peaks in a spectrum are at well‐defined $\omega_{\text{RF}}$, it is possible to measure $B_{1}^{+}$ from the difference in the Bloch‐Siegert phase shift between multiple peaks in a single spectrum (with a single Bloch‐Siegert pulse frequency), or from the totality of Bloch‐Siegert phase shifts of multiple peaks in multiple spectra (with different Bloch‐Siegert pulse frequencies for each acquisition). It is not immediately apparent whether incorporating signals from multiple peaks is beneficial when determining $B_{1}^{+}$. And it is unclear whether it is more efficient to devote the whole measurement time to a single spectrum (with maximal SNR) using one Bloch‐Siegert pulse frequency or whether it is better to acquire several spectra (with lower SNR) at several Bloch‐Siegert pulse frequencies.

To investigate, we calculated the accuracy and precision of a representative selection of measurement strategies using Monte Carlo simulations and the propagation‐of‐errors method, applied to each strategy in turn as follows:

1. Deduce an expression that computes $\gamma B_{1}^{+}$ from the measured metabolite phases, e.g., Eq. (3). (And where relevant, generalize to an arbitrary number of metabolite peaks.)
2. Estimate experimental accuracy (bias) by Monte Carlo simulation using this expression. For each peak in a simulated spectrum, generate an array of 10^6^ normally distributed phases with mean equal to the phase value from Eq. (1) (for the appropriate Bloch‐Siegert‐pulse‐to‐metabolite–frequency‐offset $\omega_{i}$ and $B_{1}^{+}$) and with standard deviation Δϕ. Express the resulting accuracy as a percentage deviation from the true $B_{1}^{+}$,
  (4) $${ɛ}_{B}=100\frac{1}{N}\sum_{i=1}^{N}(B_{1}^{i}-B_{1,\text{true}}^{+})/B_{1,\text{true}}^{+}.$$
3. Compute experimental uncertainty $\Delta \gamma B_{1}^{+}$ by the propagation of errors 20:
  (5) $$\Delta q=\sqrt{{\sum_{i}\left(\frac{\partial q}{\partial i}\times \Delta i\right)}^{2}}$$
  where ∂q/∂i is the partial derivative of q with respect to the $i^{th}$ measured variable and Δi is the uncertainty in the measurement of *i*. Express the uncertainty as the coefficient of variation in percent $(C_{v}=\;100.\Delta \gamma B_{1}^{+}/\gamma B_{1,\text{true}}^{+}).$

Each measurement strategy was considered for use in 7T cardiac ^31^P‐MRS. The simulation variables were set as follows to the study means in reference 21, because the hardware and acquisition method were identical to those used here. The PCr, γ−, α−, and β−adenosine triphosphate (ATP) peaks were simulated at frequencies of 0Hz, ‐300 Hz, ‐900 Hz, and ‐1950 Hz, respectively. Single peak simulations used only the PCr peak (at 0 Hz). The phases ϕ were calculated for a 3.5 ms Fermi pulse with $\gamma B_{1,\text{peak}}^{+}$= 277 Hz placed at $\omega_{RF}=\pm 2000\;Hz$. The phase standard deviation Δϕ was set equal to the Cramér‐Rao Lower Bound of the phase, calculated as described in Cavassila et al 22, for the study mean in Rodgers et al 21, divided by the square‐root of the number of scans per measurement ( $\text{CRLB}\varphi /\sqrt{N}$). Each measurement strategy was also evaluated for Δϕ values corresponding to the mean SNR ± standard deviation in Rodgers et al 21. The derived equations and results are given in Table 1.

**Table 1:** Comparison of Possible Bloch‐Siegert Spectroscopy $B_{1}^{+}$ Measurement Strategies Applied to ^31^P‐MRS^a^

Measurement strategy	Peaks (^31^P equivalent)	Scan 1	Scan 2	$B_{1}^{+}$ Determination equation $\gamma B_{1,\text{peak}}^{+}$	Propagation of error $\Delta \gamma B_{1,\text{peak}}^{+}$	Bias ± SD (Hz) SNR = 18 (μ‐1σ)	Bias ± SD (Hz) SNR = 31 (μ)	Bias ± SD (Hz) SNR = 44 (μ+1σ)
A: Dual‐acquisition +/‐	1 (PCr)	3.5ms Fermi +2000Hz	3.5ms Fermi −2000Hz	$\sqrt{\frac{(\phi_{2}-\phi_{1})\omega_{\text{RF}}}{2\pi \tilde{B}}}$	$\sqrt{\frac{\omega (\Delta \phi_{+}^{2}+\Delta \phi_{-}^{2})}{8\pi (\phi_{+}-\phi_{-})\tilde{B}}}$	2.21 ± 53.7	0.52 ± 27.9	0.28 ± 21.5
B′: Dual‐ acquisition on/off	1 (PCr)	3.5ms Fermi +2000Hz	No Bloch‐Siegert pulse	$\sqrt{\frac{(\phi_{\text{on}}-\phi_{\text{off}})\omega_{\text{RF}}}{\pi \tilde{B}}}$	$\sqrt{\frac{\omega (\Delta \phi_{\text{on}}^{2}+\Delta \phi_{\text{off}}^{2})}{4\pi (\phi_{\text{on}}-\phi_{\text{off}})\tilde{B}}}$	8.19 ± 76.0	2.44 ± 39.5	1.32 ± 30.3
B″: Dual‐ acquisition on/off	4 (PCr, γ‐ATP, α‐ATP, β‐ATP)	3.5ms Fermi +2000Hz	No Bloch‐Siegert pulse	$\frac{\sum_{i=1}^{n}\pi^{-\frac{1}{2}}\omega_{i}^{-2}\sqrt{\frac{(\phi_{i}^{on}-\phi_{i}^{off})\omega_{i}}{\tilde{B}}}}{\sum_{i=1}^{n}\omega_{i}^{-2}}$	$\sqrt{\sum_{i=1}^{n}\frac{{\left(\Delta \phi_{i}^{\text{on}}\right)}^{2}+{\left(\Delta \phi_{i}^{\text{off}}\right)}^{2}}{4\pi \tilde{B}{\left(\sum_{i=1}^{n}\omega_{i}^{-1}\right)}^{2}\left(\phi_{i}^{\text{on}}+\phi_{i}^{\text{off}}\right)\omega_{i}}}$	9.03 ± 70.1	3.97 ± 36.5	2.27 ± 28.0
C: Single‐ acquisition	4 (PCr, γ‐ATP, α‐ATP, β‐ATP)	3.5ms Fermi +2000Hz	No scan	$\frac{\sum_{i=2,j=1}^{n,i-1}\frac{\frac{\sqrt{\phi_{j}-\phi_{i}}}{\tilde{B}}{\left(\omega_{j}^{-1}-\omega_{i}^{-1}\right)}^{2}}{\sqrt{\pi }\sqrt{\omega_{j}^{-1}-\omega_{i}^{-1}}}}{{\sum_{i=2,j=1}^{n,i-1}\left(\omega_{j}^{-1}-\omega_{i}^{-1}\right)}^{2}}$	b	8.67 ± 153.8	7.34 ± 83.1	5.58 ± 63.9

a A fuller description of the methods are given in the multi‐peak approaches subsection of Theory. The values used to calculate the bias and standard deviation are taken from the study means of ref 21, the bias is calculated using Eq. (4) with $\gamma B_{1,\text{true}}^{+}=\;277\text{Hz}$.

b A generalized closed form could not be found for the uncertainty, $\Delta \gamma B_{1,\text{peak}}^{+}$, of Method C (the single‐acquisition method), however an expression may be found by expanding the equation for determining $B_{1}^{+}$ for a specific number of peaks and then applying Eq. (5). We provide a Mathematica notebook in the supporting information, for the four peak case that could easily be adapted for other situations.

### Method A: Dual‐Acquisition +/−

This approach is equivalent to the original Bloch‐Siegert $B_{1}^{+}$‐mapping MRI technique 15. Two acquisitions are made with Bloch‐Siegert pulses at offsets symmetrically either side of the target metabolite. This approach computes $B_{1}^{+}$ using signal from a single peak.

### Method B: Dual‐Acquisition On/Off

#### Single‐peak (B′)

This approach uses one acquisition without a Bloch‐Siegert pulse and one acquisition with the Bloch‐Siegert pulse on one side of the resonances. $B_{1}^{+}$ is computed from a single peak.

#### Multi‐peak (B″)

This acquisition also allows simultaneous use of data from all peaks that satisfy the linearity condition: $\gamma B_{1,\text{peak}}^{+}\ll |\omega_{i}|$. (N.B. each peak may have a positive or negative offset.) We combine the measured $B_{1}^{+}$ values from each resonance peak in a maximum likelihood sense, which is equivalent to the outcome that would be achieved from least‐squares fitting of the linear Eq. (1) to the simulation data. The equations generated can be expressed for any number of resonances n at offsets $\omega_{i}$ from the Bloch‐Siegert pulse.

### Method C: Single‐Acquisition Method

Measurement of $B_{1}^{+}$ from a single Bloch‐Siegert sensitized acquisition may be achieved for a multipeak spectrum, providing the first‐order phase correction and excitation phase profile are known. For *n* peaks, a single acquisition is made with a Bloch‐Siegert pulse placed so that the linearity condition is satisfied for all the peaks to be included in the measurement. To calculate $B_{1}^{+}$ Eq. (2) is applied to the phase differences between each pair of peaks and this set of $B_{1}^{+}$ values may be averaged in a maximum‐likelihood sense.

### Bloch‐Siegert Pulse Design

An ideal Bloch‐Siegert pulse maximizes $\phi_{\text{BS}}$, for some anticipated range of $B_{1}^{+}$, at the same time minimizing direct excitation, and minimizing signal loss due to any additional dead‐time between excitation and readout (when the transverse magnetization experiences T_2_^*^ relaxation). Providing one remains in the linear regime ( $\omega_{RF}\gg \gamma B_{1}^{+},\text{maximum}\;\;\gamma B_{1}^{+}\approx 1200\;Hz$) and assuming that the SAR contribution from the excitation pulse is negligible, Eq. (6) in Duan et al 18 shows that the Bloch‐Siegert phase difference is limited only by the SAR limit. Therefore, an optimized pulse must be chosen by minimizing direct excitation at the target frequency offset and by minimizing T_2_^*^‐induced signal losses.

A commonly employed Bloch‐Siegert pulse is the Fermi pulse. We use the definition in Eq. [4.14] of Bernstein et al 19:

(6) $$B_{1}^{+}(t)=\frac{A_{F}\text{exp}(i\omega_{\text{RF}}t)}{1+\text{exp}[(|t|-T_{0})/a]}$$

where $A_{F}$ is the $B_{1}^{+}$‐field peak amplitude, $\omega_{\text{RF}}$ is the pulse frequency offset and $T_{0}$ and a are two adjustable parameters having the dimension of time.

## METHODS

All experiments used a Magnetom 7T scanner (Siemens, Erlangen, Germany). Localizers were acquired with a 10 cm ^1^H loop coil (Rapid Biomedical, Rimpar, Germany). This was replaced by a T/R switch and preamplifier module (Virtumed LLC, MN, USA) connected to a 10‐cm ^31^P transmit–receive loop coil 21 for the ^31^P acquisitions. The ^31^P coil was tuned and matched using an RF sweeper (Morris Instruments Inc, Ottawa, Canada) for each subject. All subjects in this study were recruited in a manner approved by the local Research Ethics Committee.

### Fermi Pulse Optimization

The suitability of a range of Fermi pulses was examined using a Bloch simulation grid search. $T_{P}\;$was varied from 1 ms to 10 ms in steps of 0.5 ms, $T_{0}$ was varied from $0.1T_{P}\;$to $0.5T_{P}\;$in steps of $0.05T_{P}\;$and a was varied from $0.01T_{P}\;$to $0.3T_{P}\;$in steps of $0.001T_{P}$. Spin evolution was simulated in Matlab (Mathworks, Natick, MA) at $\gamma B_{1}^{+}$ values ranging from 100 Hz to 1000 Hz in steps of 100 Hz. The deviation from the ideal flat stop‐band profile was quantified as

(7) $$\Delta M_{\text{xy}}=\frac{1}{1000}\sum_{\omega =1500}^{2500}|M_{\text{xy}}^{\omega }{|}^{2}.$$

The minimum $\Delta M_{\text{xy}}$of the shortest pulse which maintained a $\Delta M_{\text{xy}}<0.01$ across all simulated peak $B_{1}^{+}$ values had $T_{P}=3.5\;ms,\;T_{0}=0.875\;ms,\;\text{and}\;a=0.224\;\text{ms}$. These parameters were used for all experiments presented below.

To assist in the implementation of the Bloch‐Siegert method and pulse optimization step, we provide the Matlab implementation of this optimization in the file “FermiPulseOptimisation.m” in the Supporting Information, which is available online.

### Point‐Source Phantom Validation

**Figure 1:**
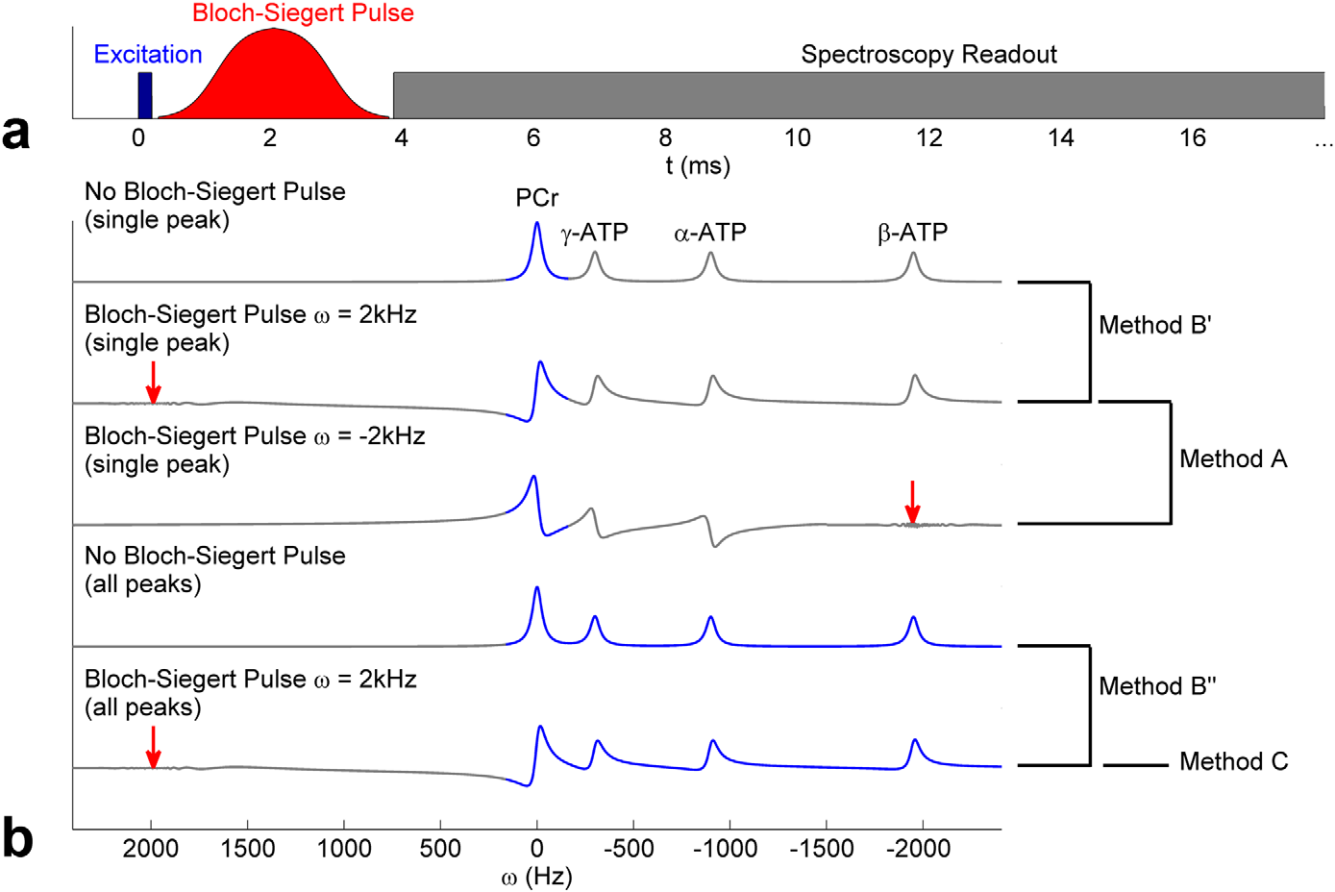
**a:** Pulse sequence timing diagram for a Bloch‐Siegert spectroscopy $B_{1}^{+}$‐mapping sequence. The Bloch‐Siegert sensitizing pulse is inserted between the excitation pulse and the readout module. In this work, the readout module consists of 3D phase encoding gradients, acquisition of a free induction decay, spoiler gradients and a final delay to produce the desired TR. The sequence is adapted from that in Figure 1 of reference 21. **b:** Illustration of the real part of ^31^P spectra acquired for each of the measurement strategies detailed in the Theory Section and corresponding to a row in Table 1. The peaks used for analysis in each case are shown in blue. The position of the Bloch‐Siegert pulses are shown by red arrows. The phase accumulated by each peak is proportional to the inverse of its frequency offset from the Bloch‐Siegert pulse (Eq. (1)). Note that when the Bloch‐Siegert pulse is placed at ‐2 kHz, the β‐ATP peak is almost entirely saturated.

The Bloch‐Siegert effect was demonstrated in a saline‐filled (18 L of 73 mM NaCl) phantom containing a single, small cube (2 × 2 × 2 cm^3^), of 1.8 M K_2_HPO_4_, which gives one singlet signal that originates only from the cube. The optimized Fermi pulse ( $T_{P}=3.5\;ms,\;T_{0}=0.875\;ms,\;a=0.224\;\text{ms})$ was inserted between the excitation RF pulse and the 3D phase‐encoding gradients of a chemical shift imaging (CSI) sequence (Fig. 1a) 21, 23. The Fermi pulse offset was swept from ‐10 kHz to +10 kHz.

Acquisition parameters were: 4 × 4 × 4 CSI matrix, 80 × 80 × 80 mm^3^ field of view, 2048 spectral points, 6 kHz bandwidth, 200 μs hard excitation pulse with a transmit voltage, i.e., the RMS voltage at the “coil plug” on the patient table, of 270 V. The voxel covering the 2 × 2 × 2 cm^3^ phosphate cube was selected for analysis. Spectra were fitted using a custom Matlab implementation of the advanced method for accurate, robust and efficient spectral fitting (AMARES) algorithm 24, with prior knowledge specifying a single unconstrained peak 25.

A $\gamma B_{1,peak}^{+}$ value was measured using a series of fully relaxed, nonlocalized free induction decays (FIDs) with a 4‐ms hard pulse transmitted at 10–200 V. Under these conditions, where $T_{R}>5T_{1}$, the amplitude of the observed FIDs will follow a sinα relationship. The FIDs were fitted in Matlab, and then a sinusoid was fitted to the complex peak amplitudes with two adjustable parameters: the maximum signal amplitude, and the $B_{1}^{+}$‐per‐volt scaling factor that relates the (known) applied transmit voltage to the observed signal amplitudes (i.e. flip angle). $\gamma B_{1,peak}^{+}$ may be calculated as the product of the scaling factor and the Bloch‐Siegert pulse transmit voltage and subsequently used to predict the phase response for the Bloch‐Siegert pulse.

### Uniform Phantom Validation

Bloch‐Siegert $B_{1}^{+}$‐mapping was validated against a multi‐TR magnitude reference method in a uniform phantom. The phantom was a 120 × 270 × 270 mm^3^ box containing 0.04 M K_2_PO_4(aq)_, giving rise to a singlet signal from throughout the whole volume of the phantom. The T_1_ of the phantom was separately determined to be 8.57 s. The acquisition parameters were: 300 μs hard pulse excitation transmitted at 270V, 6 kHz bandwidth, 2048 spectral points, 150 × 320 × 320 mm^3^ field of view, acquisition weighting, 16 × 8 × 8 resolution, the first dimension perpendicular to the plane of the coil. The multi‐TR method was adapted from a previously published dual‐TR method 11, acquiring spectra at eight TRs (0.5, 1, 2, 3, 4, 6, 8, 10 s), to ensure functional sensitivity to a broad range of flip angles simultaneously. The number of averages at k = 0 was adjusted for each acquisition, to achieve similar high SNR for a “best possible” validation; acquisitions times were between 80 and 120 min each (giving a total of 12.5 h run overnight). The partial saturation equation,

(8) $$\frac{S(\theta ,T_{R},T_{1})}{M_{0}}=\frac{\text{sin}(\theta )\cdot (1-\text{exp}(-T_{R}/T_{1}))}{\left(1-\text{exp}(-T_{R}/T_{1})\cdot \text{cos}(\theta )\right)}$$

was fitted to the magnitude data to determine the flip angle, $\gamma B_{1}^{+}$ was calculated from the relationship $\theta =\;180{}^{\circ}.T_{P}.\gamma B_{1}^{+}/500\text{Hz}$ where $T_{P}$ is the duration of the hard excitation pulse.

Bloch‐Siegert acquisitions used the same parameters, with the optimized Fermi pulse ( $T_{P}=3.5ms,\;T_{0}=0.875\;ms,\;a=0.224)$ inserted as in Figure 1a. Two acquisitions were made with the pulse placed at +2 kHz and ‐2 kHz offsets. TR was 500 ms, with 13 averages at k = 0, resulting in a total duration of 2 × 14 min.

To avoid artifacts due to phase‐wrap present in the raw phase maps, phase differences were calculated using complex division and the Matlab function atan2() that returns phase between −π and π,

(9) $$\Delta \phi =\phi_{1}-\phi_{1}={\text{atan2}[\text{Im(e}}^{i\phi_{1}}/e^{i\phi_{2}}),\text{Re}(e^{i\phi_{1}}/e^{i\phi_{2}})]$$

then $\gamma B_{1}^{+}$ was computed using Eq. (3). Voxels with Δϕ<0º (indicating either very low $\gamma B_{1}^{+}$ or the presence of uncorrected wrap artefacts in the phase difference map, i.e., true Δϕ>180º), voxels outside the phantom, or voxels with a $CRLB_{\phi }>20{}^{\circ}$ (indicating low SNR) were excluded.

### In Vivo Validation (Thigh)

Full details of the acquisition strategy analysis are given in “Simulation results” below. Method A (dual‐acquisition +/−) showed highest precision and accuracy and was used for all measurements in this work.

Bloch‐Siegert $B_{1}^{+}$‐mapping was compared with a published dual‐TR flip‐angle mapping method 11 in a healthy volunteer's quadriceps (male, 28 years). Acquisition parameters were: 300 μs hard pulse excitation transmitted at 270 V, 200 × 200 × 200 mm^3^ field of view, 16 × 8 × 8 resolution (16 perpendicular to the coil), acquisition weighting, 6 kHz bandwidth and 2048 spectral samples.

In the dual‐TR comparison, excitation was centered on α‐ATP (rather than PCr), to avoid complications due to the creatine‐kinase mediated exchange of PCr and γ‐ATP 6. A literature value of $T_{1,\alpha -ATP}$ = 1.8 s was used to calculate the flip‐angle according to the published method 26. Three acquisitions were made with TRs of 2.2 s, 0.73 s, and 0.37 s, using 10, 20, and 43 averages at the center of k‐space for acquisition times of 25, 15, and 15 min, respectively, as recommended in Chmelik et al 11.

Two Bloch‐Siegert acquisitions were made with the optimized Fermi pulse ( $T_{P}=3.5\;ms,\;T_{0}=0.875\;ms,a=0.224)\;$placed at +2 kHz and ‐2 kHz relative to PCr; excitation was centered at PCr. TR was 500 ms, with 25 averages at k = 0, resulting in a total duration of 2 × 12.5 min.

Fitting was performed with AMARES in Matlab using prior knowledge specifying 10 Lorentzian peaks (α,β,γ‐ATP multiplet components, PCr, phosphodiesters [PDE], and inorganic phosphate [P_i_]), with fixed amplitude ratios and scalar couplings for each multiplet. $\gamma B_{1}^{+}$ was calculated as described in “Uniform phantom validation” above. Voxels were excluded from the comparison if the voxel was centered outside the thigh, Δϕ<0º or CRLB_ϕ_>10^°^.

### Cardiac Scans

Cardiac ^31^P Bloch‐Siegert $B_{1}^{+}$‐mapping was demonstrated in five healthy subjects (male, 25–46 years, 75–85 kg, 1.79–1.93 m). The scan protocol followed Rodgers et al 21, with subjects positioned head‐first supine.

Two Bloch‐Siegert acquisitions were made with the optimized Fermi pulse ( $T_{P}=3.5\;ms,\;T_{0}=0.875\;ms,a=0.224)\;$placed at +2 kHz and ‐2 kHz from PCr, and excitation by a 200 μs hard pulse centered on PCr. The excitation pulse voltage was set to achieve a flip angle of 30^°^ at the mid‐interventricular septum [the Ernst angle for a $T_{R}=500\;ms$ and a PCr $T_{1}=\;3.09\;s$ 21]. Acquisition parameters were: 240 × 240 × 200 mm^3^ field of view, 16 × 8 × 8 resolution (16 perpendicular to the coil), acquisition weighting with 20 averages at k = 0, 6 kHz bandwidth and 2048 spectral samples. Total scan time was 2 × 10.5 min.

Fitting was performed with AMARES in Matlab using prior knowledge specifying 11 Lorentzian peaks (α,β,γ‐ATP multiplet components, PCr, PDE, and the two peaks of 2,3‐diphosphoglyceric acid [DPG]), with fixed amplitude ratios and scalar couplings for each multiplet. $\gamma B_{1}^{+}$ was calculated as described in “Uniform phantom validation” above. Voxels were excluded if Δϕ<0º or CRLB_ϕ_>15°. Phase wrap, caused by the upper output limit of Eq. (9) was unwrapped manually.

In three subjects, the scans were repeated with the central frequency adjusted to α‐ATP to allow comparison of $\gamma B_{1}^{+}\;$values obtained using different peaks.

## RESULTS

### Simulation Results

**Figure 2:**
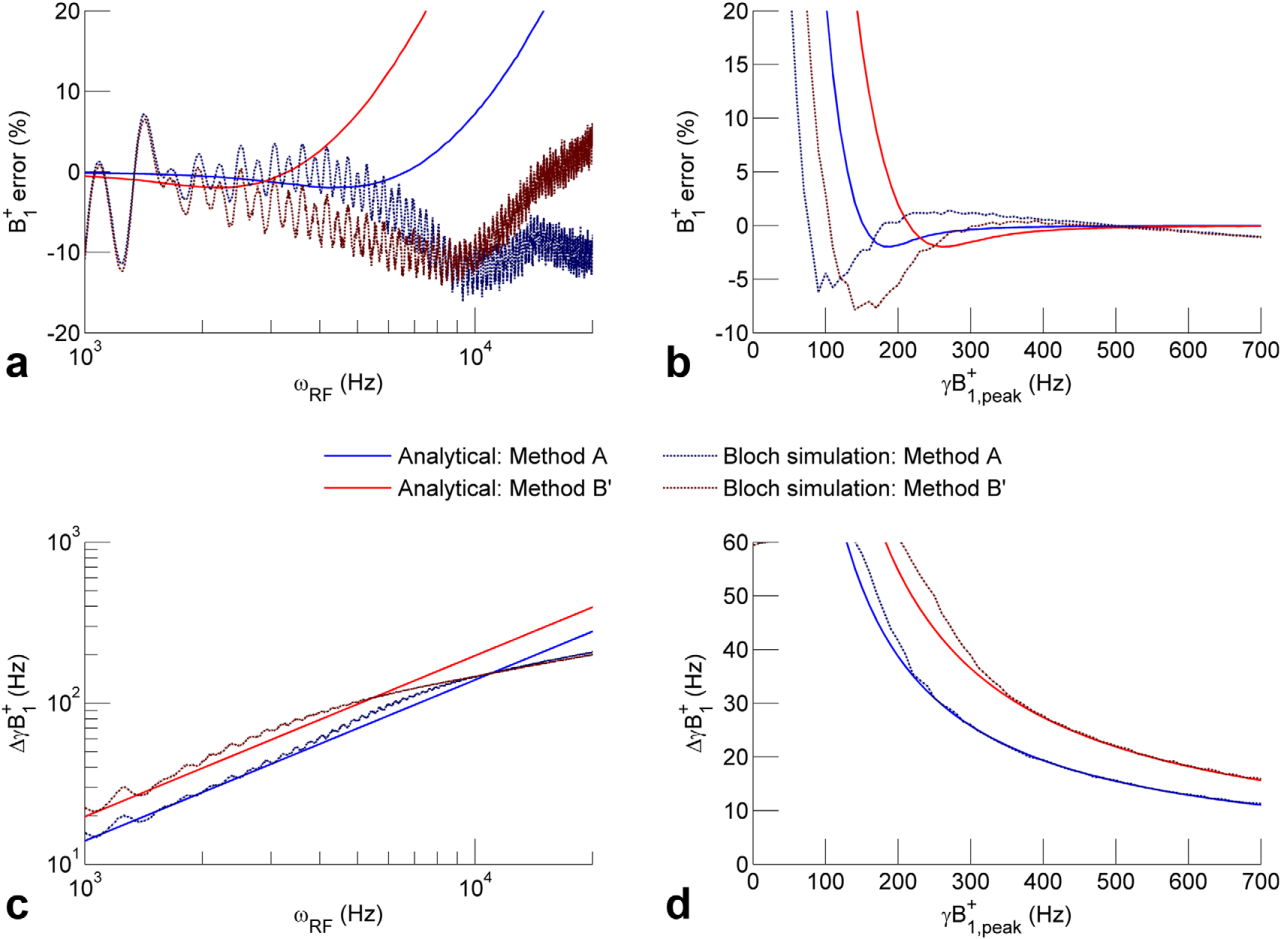
Precision and accuracy of the single‐peak acquisition strategies [Method A (blue line) and Method B′ (red line)] computed from the analytical expressions in Table 1. A numerical Bloch simulation of both methods, with the optimized Fermi pulse, is shown for comparison (dashed lines). Note that the differences apparent in the Bloch simulations in panel (a) and (b) arise due to direct excitation and correspond to the oscillations in $B_{1}^{+}$ fractional error in Figure 4c. The magnitude of the bias introduced scales with the simulation $\gamma B_{1}^{+}$ (277Hz). The standard deviation calculated analytically is not affected by direct excitation. **a:** The fractional error, as defined by Eq. (4) of the simulated mean from the true $\gamma B_{1}^{+}$ (277Hz) as a function of Fermi pulse offset from the central frequency $\omega_{\text{RF}}$. **b:** The fractional error from $\gamma B_{1,\text{true}}^{+}$ as a function of $\gamma B_{1,\text{peak}}^{+}$. **c,d:** Standard deviation of $\gamma B_{1}^{+},{\;\Delta \gamma B}_{1}^{+}$ as a function of pulse offset and $\gamma B_{1,\text{peak}}^{+}$.

The precision and accuracy of the single‐peak measurement strategies (A and B′) are shown in Figure 2. Methods A and B′ show <10% error and <10% uncertainty for $1000\;\text{Hz}<\omega_{\text{RF}}<3000\;\text{Hz}$ and $\gamma B_{1}^{+}>150\;\text{Hz}$. Method B′ has both precision and accuracy $\sqrt{2}$ worse than Method A . These calculations do not take into account the effects of direct excitation, which is shown in the Bloch simulations. The error caused by the direct excitation is generally larger than the underlying error arising from Δϕ, emphasizing the importance of effective pulse design.

**Figure 3:**
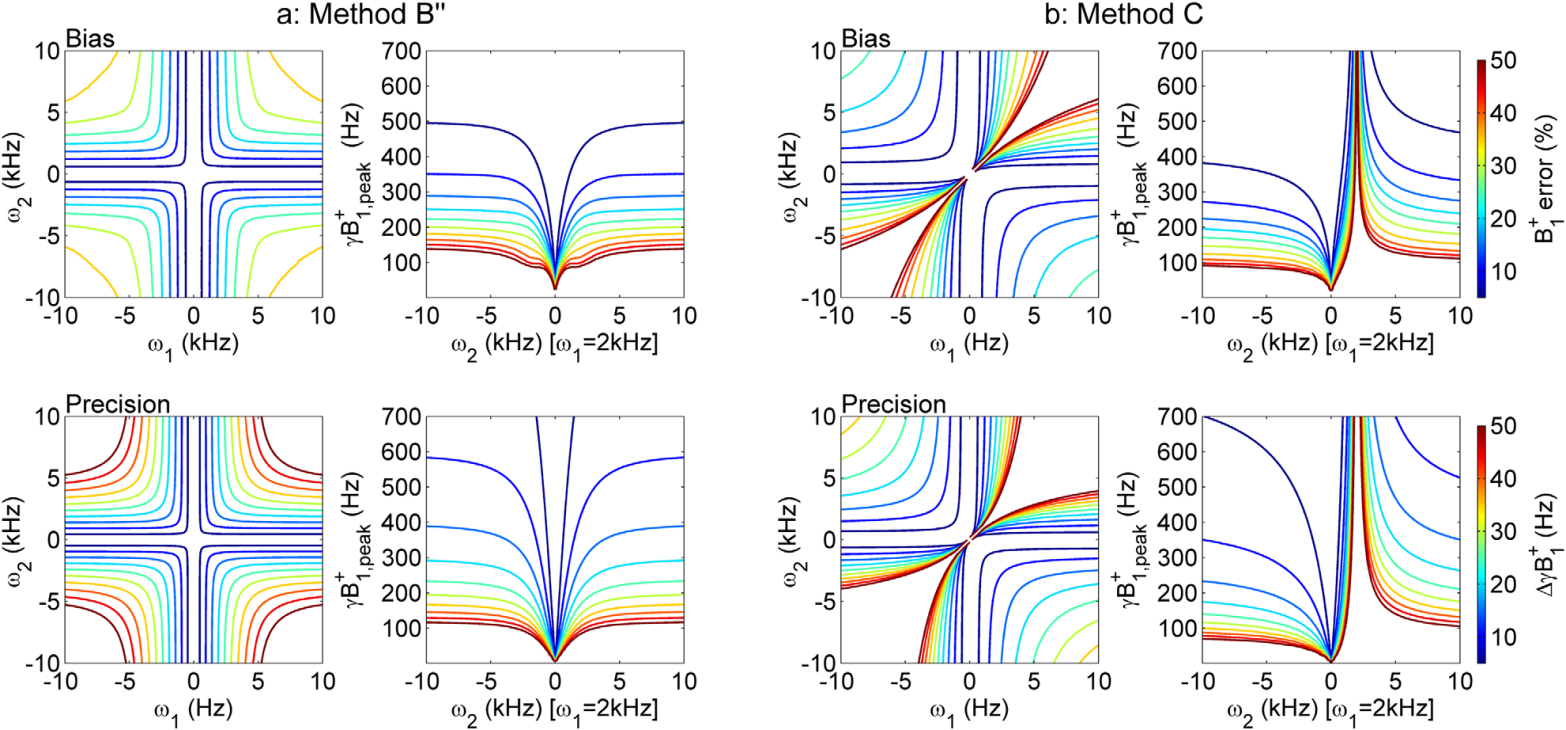
Fractional $B_{1}^{+}\;$error (Eq. (4)), “Bias”, and standard deviation, “Precision”, of the multipeak acquisition strategies for a two peak spectrum. **a:** Method B″. **b:** Method C. The error and standard deviation are plotted as functions of the individual peak offsets from the Bloch‐Siegert sensitizing pulse, $\omega_{1}$and $\omega_{2}$; and as the second peak position $\omega_{2}$ (with $\omega_{1}$ fixed at 2 kHz) and $\gamma B_{1,\text{peak}}^{+}$. The calculations do not take into account any direct excitation caused by the Bloch‐Siegert sensitizing (Fermi) pulse, therefore, the optimum separation will be a compromise between minimizing excitation and minimizing error and standard deviation. Method C (multipeak single‐acquisition) shows a $\sqrt{2}$ improvement over Method A when the Bloch‐Siegert pulse can be placed symmetrically between the peaks. Method C has worse performance than Method B″ if the peaks are on the same side of the Bloch‐Siegert pulse, while Method B″ is independent of the sign of $\omega_{i}$.

The analysis of the multi‐peak methods (B″ and C) results in smoothly varying functions (Fig. 3). Increasing $\gamma B_{1,\text{peak}}^{+}$ always improves the precision and accuracy. In Figure 3a (Method B″) minimizing $\left|\omega_{i}\right|$, maximizes $\phi_{\text{BS}}$ and improves the precision and accuracy, while adding additional peaks ( $\omega_{2,3,\ldots })$ with a larger $\left|\omega_{\text{RF}}\right|$ has only a small effect. In Figure 3c, Method C shows lower accuracy and higher bias than the other methods when all the offsets have the same sign. However, if the Bloch‐Siegert pulse is placed symmetrically between two peaks, we calculate an identical profile to Method A with a $\sqrt{2}$ improvement in both accuracy and precision for a matched scan duration, although this will only be possible in situations where the inter‐peak separation is sufficient to avoid direct excitation.

Table 1 summarizes the methods considered for our 7T cardiac ^31^P‐MRS application. All the measurement strategies show reasonable accuracy (<10% error) and most show an acceptable precision. Method A (dual‐acquisition +/−) shows better precision and accuracy than any other method (0.2% error and 10% standard deviation). Methods B″ and C, were simulated with only positive offsets from the Bloch‐Siegert pulse for our specific cardiac application. The greatest peak separation (PCr to β‐ATP) in 7T ^31^P‐MRS is 1950 Hz, which is smaller than the separation required to maintain acceptable levels of direct excitation using the optimized 3.5 ms Fermi pulse. In Bloch simulation, using the optimized 3.5 ms pulse, a separation of 1950 Hz ( $\omega_{1}=-975\;\text{Hz},\;\omega_{2}=975\;\text{Hz}$) gives a $B_{1}^{+}$ error of 19%.

### Pulse Design

**Figure 4:**
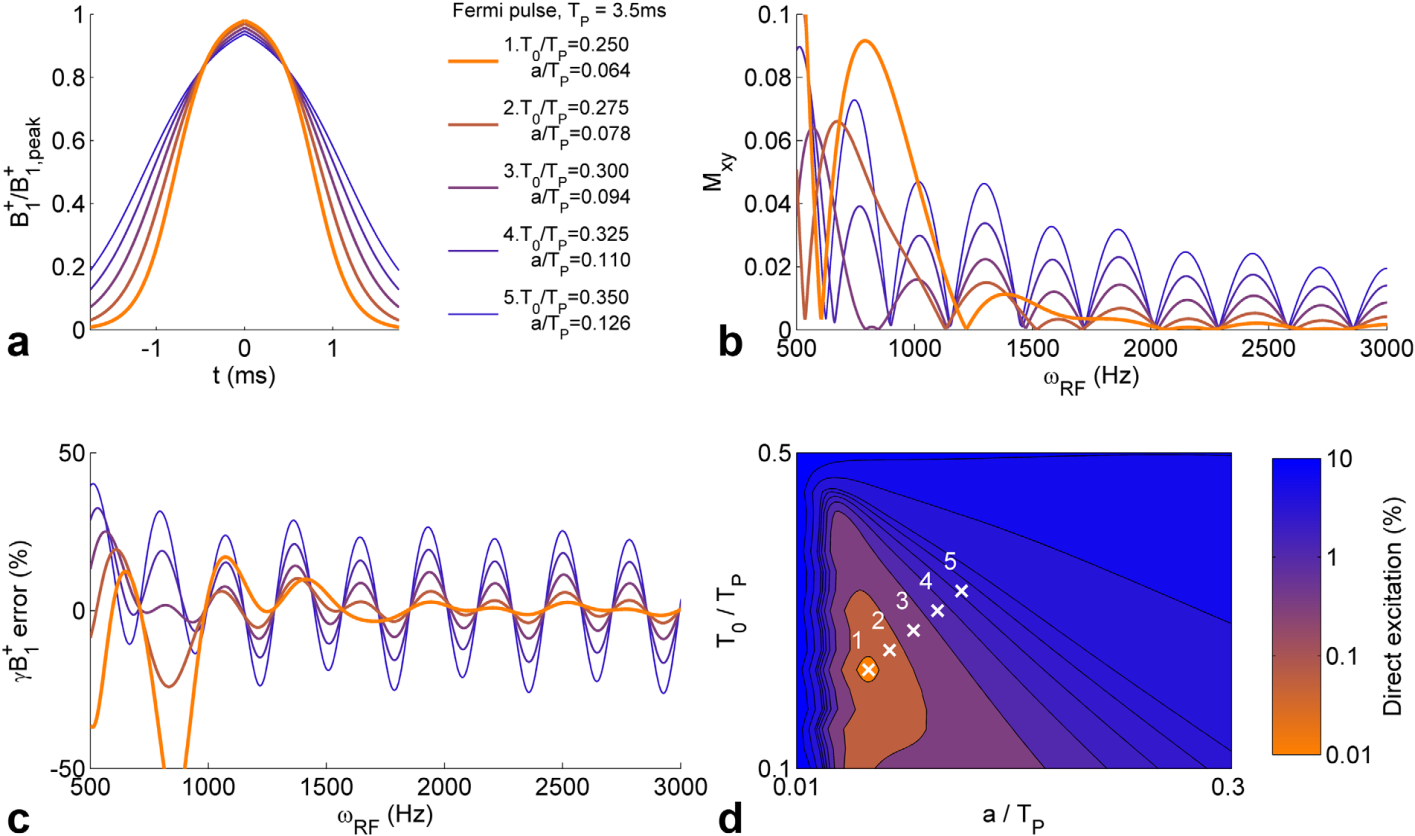
**a:** Example Fermi pulse envelopes, with fixed duration $T_{P}=3.5\;\text{ms}$, but varying the shaping parameters $T_{0}$ and a. **b:** Bloch simulation of five Fermi pulses showing the transverse magnetisation immediately after the pulse. $\gamma B_{1}^{+}$ = 1000 Hz. The orange line 1 is the pulse chosen for the experimental section of this work. **c:** Percentage error in calculation of $\gamma B_{1}^{+}$ as a result of the direct excitation caused by the Fermi pulse. **d:** Percentage deviation of the transverse magnetisation from 0 as defined by Eq. (7) for a 3.5‐ms Fermi pulse as a function of the shaping parameters $T_{0}$ and a. The “x” mark the locations of the pulses used to generate the lines in panels a–c.

The Fermi pulse selection criteria were to minimize the pulse duration whilst minimizing the direct excitation of the magnetization in the interval $1500\text{Hz}<\omega_{\text{RF}}<2500\text{Hz}$. The minimum of the direct excitation metric, $\Delta M_{\text{xy}}$ (Eq. (7)), fell below 0.01% for pulses with $T_{p}\geq 3.5\text{ms}$ (Fig. 4d). Thus the criteria, to select the shortest pulse whilst also minimizing direct excitation, were satisfied by selecting the $T_{0}\;\text{and}\;a$ parameters corresponding to the $\Delta M_{\text{xy}}$ minimum of the parameter range with $T_{P}$=3.5 ms. The resulting optimized Fermi pulse had $T_{P}=3.5\;\mathrm{ms},\;T_{0}=0.875\mathrm{ms},\;a=0.224$, and was used for all experiments and simulations in this manuscript. Figure 4 shows a range of possible Fermi pulses, the degree of direct excitation, and the $B_{1}^{+}$ error from each.

### Point‐Source Phantom Validation

**Figure 5:**
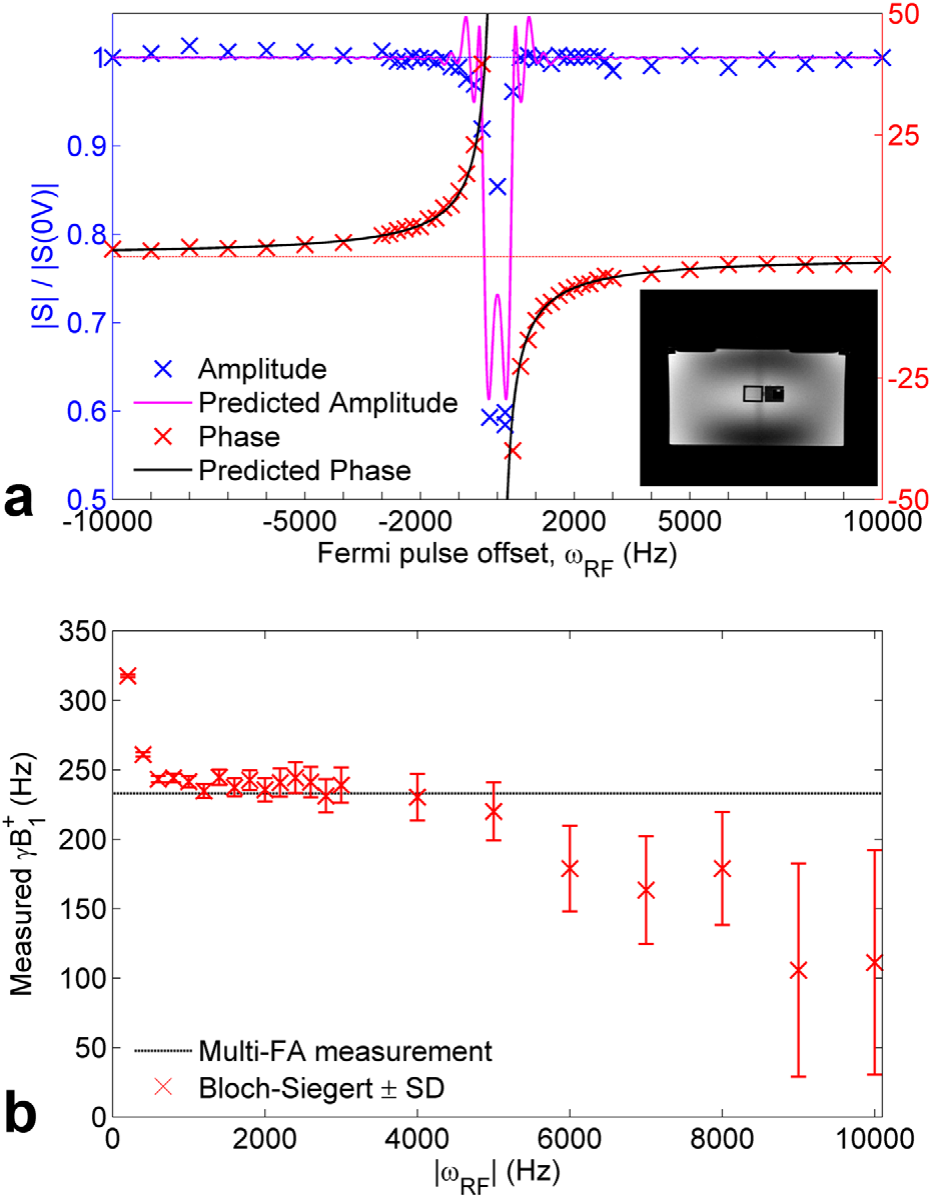
**a:** Fitted phase (red “x”) and amplitude (blue “x”) of a single peak in the presence of a Fermi pulse, as a function of Fermi‐pulse offset. The amplitude shows saturation as the narrow passband of the Fermi pulse overlaps the single phosphate peak. The phase closely follows the phase predicted by Eq. (2) using a separate $B_{1}^{+}$ measurement (solid black). The measured amplitudes are closely approximated by a Bloch simulation of the pulse sequence (magenta). The inset shows a transverse ^1^H image of the phantom, comprising a small cube of phosphate suspended in a tank of brine. **b:** $\gamma B_{1}^{+}$ calculated using the pairs of symmetric offsets from the same experiment. The values calculated by the Bloch‐Siegert method match those from a nonlocalized multi‐flip‐angle method in the range 500–4000 Hz.

The phosphate peak phase and magnitude in the single voxel phantom are displayed in Figure 5a. The predicted Bloch‐Siegert phase, calculated using the $B_{1}^{+}$ separately measured by nonlocalized, fully relaxed FIDs, and Eq. (1), and the directly measured Bloch‐Siegert phase of the single phosphate peak agree within 20% for $|\omega_{\text{RF}}|>1000\;\text{Hz}$ and within 8% for $1000\;\text{Hz}\leq |\omega_{\text{RF}}|\leq 3000\;\text{Hz}$. As $\left|\omega_{\text{RF}}\right|$increases from 1000 Hz the CRLB_ϕ_ is constant but the Bloch‐Siegert phase $\phi_{\text{BS}}$ decreases, resulting in a higher relative uncertainty. When $|\omega_{\text{RF}}|<1000\;\text{Hz}$ direct excitation of the phosphate peak by the Fermi pulse is observed, which causes the magnitude to drop substantially. For $|\omega_{RF}|>1000\;\text{Hz}$ the magnitude varies by <1.5%, indicating that direct excitation has been minimized.

Figure 5b confirms that $\gamma B_{1}^{+}$ calculated from the Bloch‐Siegert phases closely approaches that given by the Multi‐FA reference method when $500\;\text{Hz}<|\omega_{\text{RF}}|<4000\text{Hz}$.

### Uniform Phantom Validation

**Figure 6:**
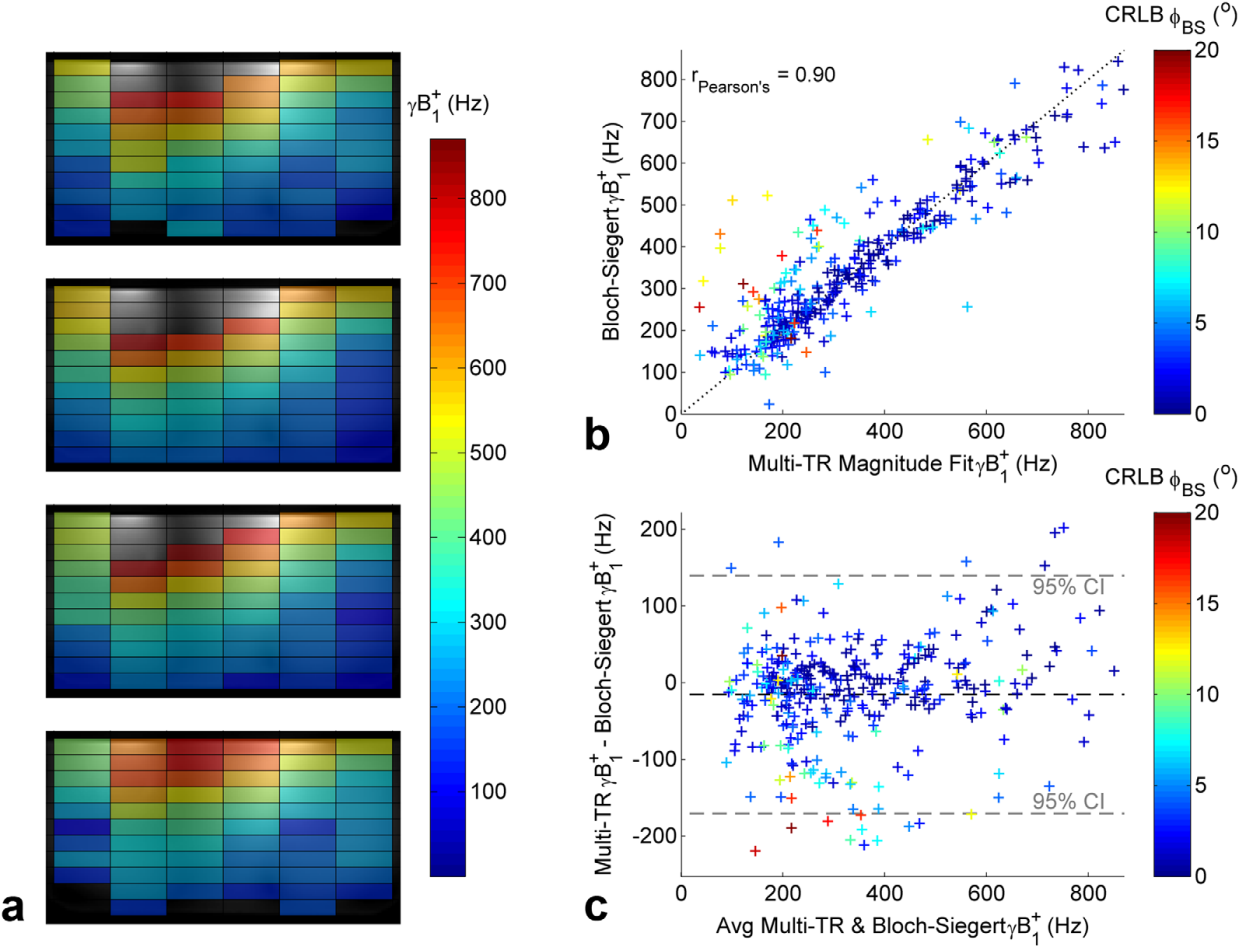
**a:** Bloch‐Siegert $B_{1}^{+}$maps, overlaid on ^1^H localizer images, across four central slices of the 16 × 8 × 8 CSI grid of the uniform phantom. Each rectangle indicates the measured value in a single ideal voxel. The maps are masked for CRLB_ϕ_, phase wrap and by the limits of the phantom. **b:** Scatter plot of the per‐voxel fitted multi‐TR validation method against the value measured by the Bloch‐Siegert method. The color indicates the CRLB_ϕ_ on the fitted phase difference and Pearson's correlation coefficient r = 0.90. **c**: Bland‐Altman plot (average of two methods versus difference) of the validation and Bloch‐Siegert methods. The color indicates the CRLB_ϕ_ on the fitted phase difference and lines show the mean difference and the ±95% confidence intervals.

Bloch‐Siegert $B_{1}^{+}$ maps across the four central CSI slices covering the uniform phantom are shown in Figure 6a. The dynamic range of the Bloch‐Siegert $\gamma B_{1}^{+}$ was 0 Hz to 873 Hz (corresponding to a π phase shift). Figures 6b,c show the per‐voxel correlation and Bland‐Altman plots of the Bloch‐Siegert method and the multi‐TR method. A strong correlation (Pearson's r = 0.90) was observed. The Bland‐Altman plot shows the Bloch‐Siegert method measured $\gamma B_{1}^{+}$ 15 Hz lower than the reference method on average, with 95% confidence intervals of ±154 Hz. The bias and confidence intervals are equivalent to ‐3.1% and 31.8%, respectively, of the $\gamma B_{1}^{+}$ value (484 Hz) of a centrally located voxel in the phantom.

### In Vivo Validation (Thigh)

**Figure 7:**
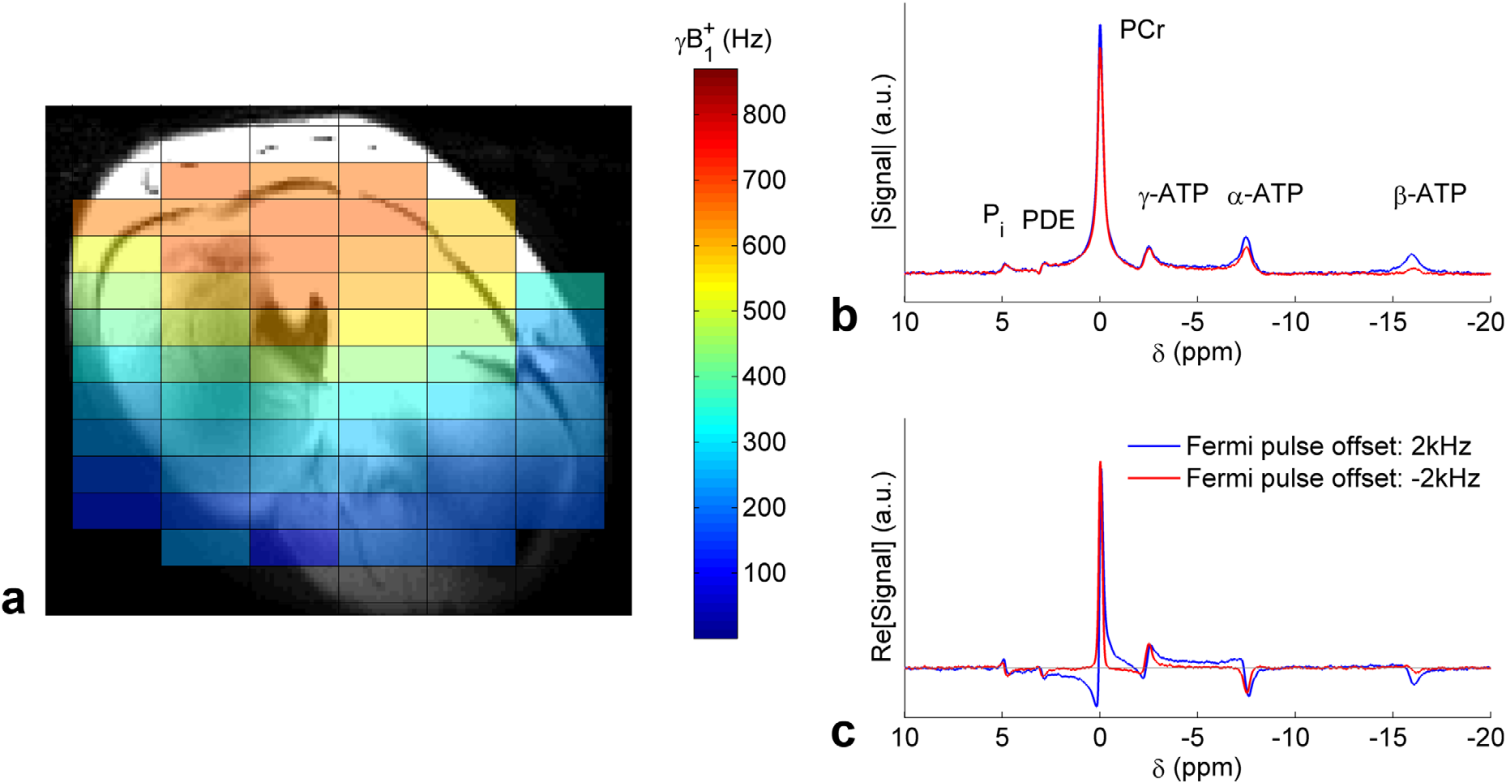
**a:** Bloch‐Siegert $B_{1}^{+}$ map from a central slice of an 16 × 8 × 8 CSI grid in a transverse plane of a healthy volunteer's quadriceps. The map is overlaid on a ^1^H localizer registered to the CSI grid. **b,c:** Example magnitude and real spectra from a central voxel in a. The maps in a are calculated from the phase of the PCr peak. The phase difference between the PCr peak of the spectrum with a 2 kHz offset (blue) on the Fermi pulse and the ‐2 kHz offset (red) is 55.6° corresponding to a value of $\gamma B_{1}^{+}$ = 484 Hz.

Figure 7 shows a Bloch‐Siegert $B_{1}^{+}$ map across a central CSI slice covering a volunteer's quadriceps and example spectra from a centrally located voxel. The Bloch‐Siegert $\gamma B_{1}^{+}$ dynamic range was 0 Hz to 873 Hz. Figures 6e,f show the per‐voxel correlation and Bland‐Altman plots of the Bloch‐Siegert and Dual‐TR methods 11. Good correlation is observed (Pearson's r = 0.84), despite the 37.5% scan time reduction from the reference to Bloch‐Siegert method. The Bland‐Altman plot shows a ‐25 Hz lower mean $\gamma B_{1}^{+}$ from the Bloch‐Siegert method compared with the Dual‐TR method, with 95% confidence intervals of ±211 Hz. The bias and confidence intervals are equivalent to ‐6.9% and 33.4%, respectively, of the $\gamma B_{1}^{+}$ value (631 Hz) of a centrally located voxel in the skeletal muscle.

### Cardiac Scans

**Figure 8:**
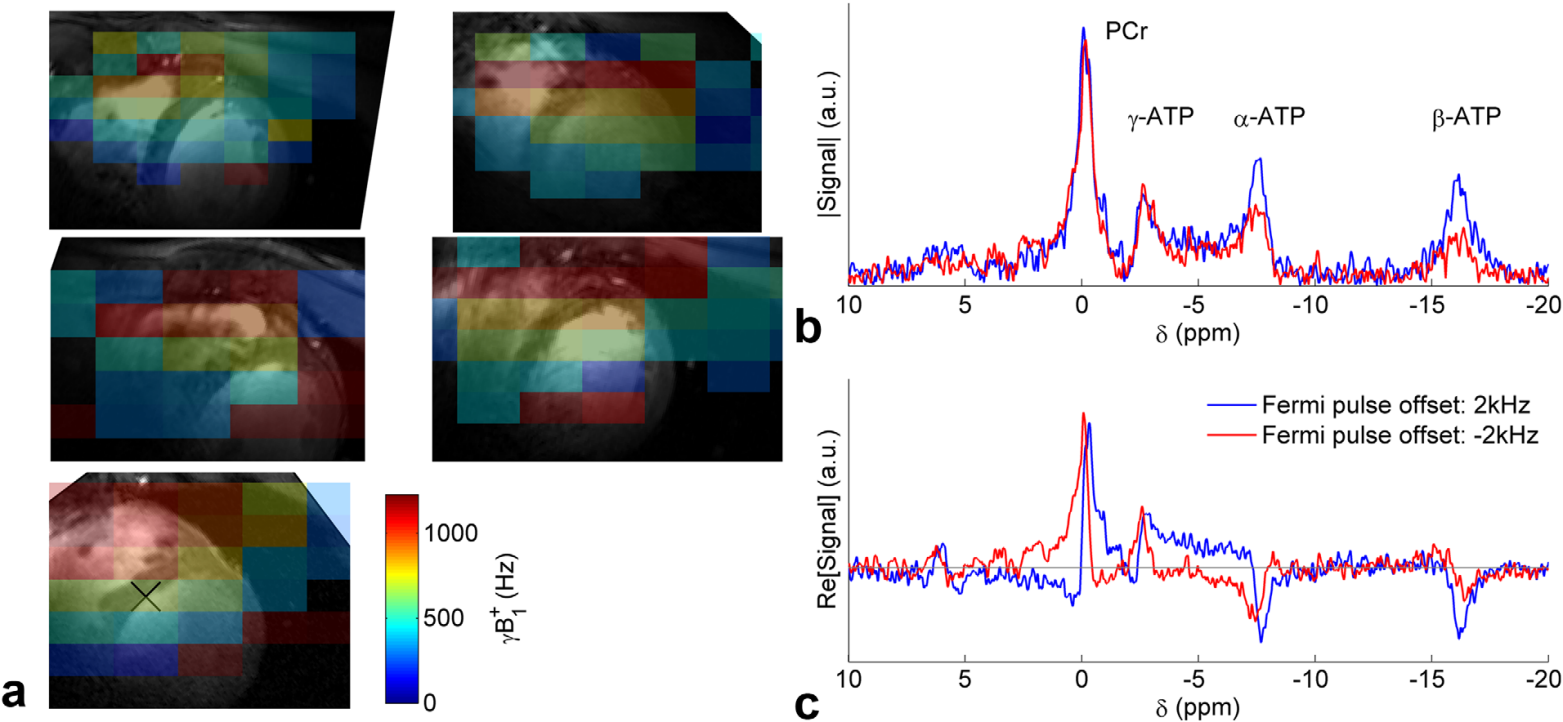
**a:** Bloch‐Siegert $B_{1}^{+}$ maps from slices of an 16 × 8 × 8 CSI grid in a mid‐short axis plane of five healthy volunteer subjects. The maps are overlaid on ^1^H localizers registered to the CSI grid. **b,c:** Example magnitude and real spectra from a mid‐interventricular septal voxel, marked by a black “x” in the lowest $B_{1}^{+}$ map in a. The maps in a are calculated from the phase of the PCr peak. The phase difference between the PCr peak of the spectrum with a 2 kHz offset (blue) on the Fermi pulse and the ‐2 kHz offset (red) is 86.9° corresponding to a value of $\gamma B_{1}^{+}$ = 605 Hz.

Five mid‐septal cardiac $\gamma B_{1}^{+}$ maps are shown overlaid on localizers in Figure 8. The Bloch‐Siegert $\gamma B_{1}^{+}$ dynamic range was 0 Hz to 1231 Hz. The short‐axis localizers were manually segmented to extract voxels in the heart and skeletal muscle in the three CSI slices corresponding to the mid, apical and basal segments of the myocardium. In the mid‐septal slices 39.6 ± 16.0 (mean ± SD) voxels were selected. After masking by CRLB_ϕ_ 30.6 ±13.7 voxels remained. The $B_{1}^{+}$ maps are smoothly varying from 100–1200 Hz without visible boundaries between skeletal muscle, myocardium and the ventricular blood pools. $B_{1}^{+}$ drops‐off with increasing distance from the coil. Data for the adjacent apical and basal slices, after masking by CRLB_ϕ_, had 28 ± 10.1 and 21.6 ± 15.6 voxels suitable for analysis, respectively, which were used in the subsequent comparisons.

**Figure 9:**
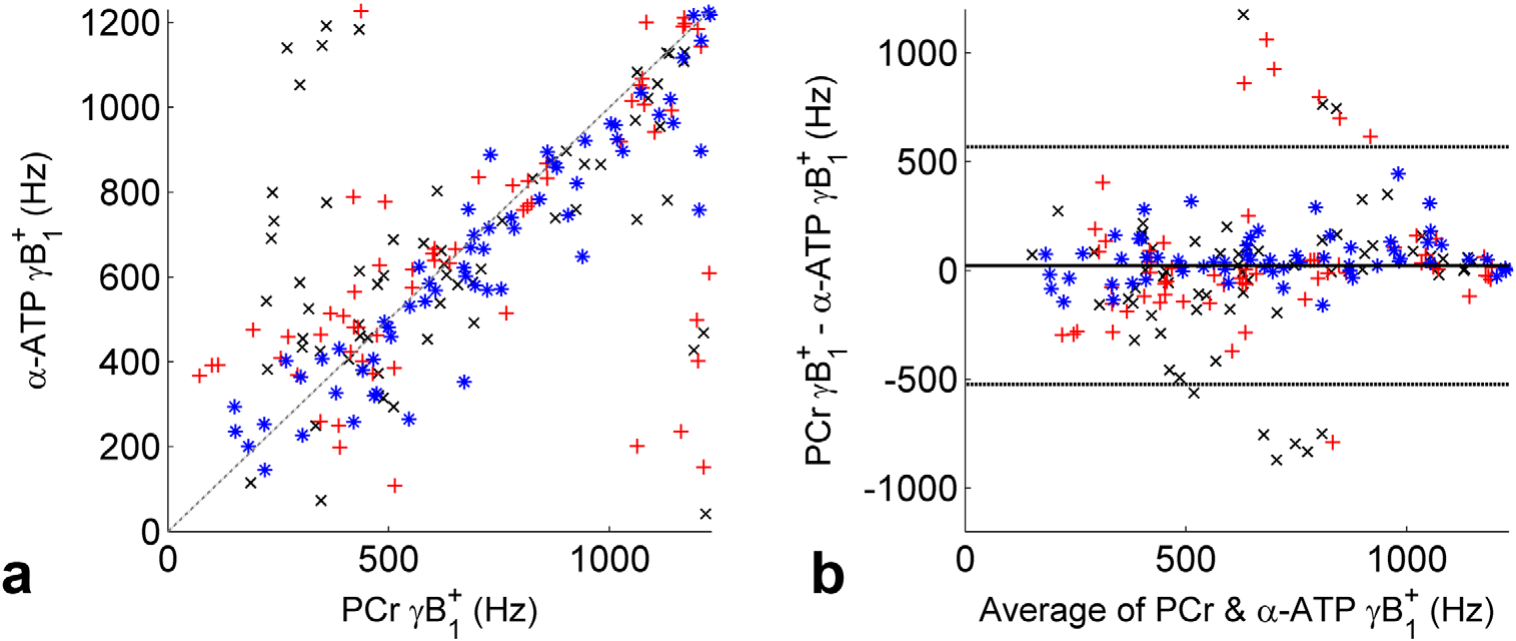
**a:** Three volunteer comparison of consecutive Bloch‐Siegert $B_{1}^{+}$ maps, collected with the Fermi pulse placed symmetrically around the PCr peak and then subsequently the α‐ATP peak in three of the subjects in the study. **b:** Bland‐Altman plot of the metabolite comparison. The means and 95% confidence intervals are calculated from all points shown.

Bloch‐Siegert measurement using α‐ATP instead of PCr is compared in Figures 9a,b. The mean Pearson's correlation was 0.62. The Bland‐Altman plot shows a bias of 22.3 Hz with 95% confidence intervals of ±561 Hz. This result confirms the T_1_‐independence found in Sacolick et al 15 and is an indication that the $B_{1}^{+}$ values measured in this work are correct.

## DISCUSSION

In this study, we proposed a Bloch‐Siegert spectroscopy $B_{1}^{+}$‐mapping technique suitable for ^31^P‐MRS in the human heart at 7T. It is straightforward to register the $B_{1}^{+}$ maps with other ^31^P scans using the same 3D CSI readout.

### T_1_ Insensitivity

Bloch‐Siegert methods are known to be T_1_‐insensitive 15. We verified this for our ^31^P‐MRS method by comparing $B_{1}$ maps derived from PCr (*T*_1_ = 3.09s) and α‐ATP (*T*_1_ = 1.39 s) in three normal volunteers. This is an advantage over multi‐TR and dual‐angle methods, which rely on metabolite $T_{1}$ values to calculate $B_{1}^{+}$.

In normal volunteers, one could use cardiac ^31^P T_1_ values from the literature in a multi‐TR or dual‐angle method 21. Yet, in studies of patients with cardiac disease, or in protocols involving exercise or pharmacological stress, these T_1_ values may be different, as has been reported for other nuclei 27. Recording 3D‐localized myocardial T_1_ values at 7T took 45 min in Rodgers et al 21—too long to be a calibration step in patients.

The problem of T_1_‐changes in disease is particularly acute for the highest SNR peak: PCr. Chemical exchange through the creatine‐kinase shuttle dominates the apparent T_1_s of PCr and γ‐ATP, 6 and the forward rate constant for this process changes markedly in disease 28. Therefore, T_1_‐sensitive $B_{1}^{+}$‐mapping methods should only be applied to nonexchanging peaks such as α‐ATP or β‐ATP, but these peaks have lower SNR than PCr. In contrast, exchange mediated phase effects during the short (3.5 ms) Fermi pulse are predicted to be <0.1% by Bloch‐McConnell simulation (not shown). Consequently, Bloch‐Siegert methods can be applied to the highest SNR peak: PCr.

The Bloch‐Siegert shift is independent of the excitation pulse or TR of the host sequence 15. Therefore, a Bloch‐Siegert $B_{1}^{+}$‐mapping sequence can be run with the Ernst flip‐angle 29 and a short TR to maximize its SNR efficiency (i.e., SNR/ $\sqrt{t})$. Bloch‐Siegert ^1^H MRI $B_{1}^{+}$‐mapping was shown to be more SNR‐efficient than dual‐angle $B_{1}^{+}$‐mapping 30. Theory predicts a similar SNR‐efficiency advantage with ^31^P‐MRS.

We, therefore, believe that Bloch‐Siegert $B_{1}^{+}$‐mapping approaches are the only known viable methods for human cardiac ^31^P $B_{1}^{+}$‐mapping at 7T.

### Bloch‐Siegert Sampling Strategy

The framework to assess spectroscopic Bloch‐Siegert measurements established that the dual‐acquisition approach is optimal for cardiac ^31^P‐MRS, because there is insufficient frequency separation to place the Fermi pulse between PCr and β‐ATP without creating artefacts due to direct excitation. In a situation where the peak separation is much wider (4000 Hz) or where T_2_* is sufficiently long to permit longer, narrower bandwidth Bloch‐Siegert sensitizing pulses, placing the pulse between two peaks will give an improvement in accuracy and precision of $\sqrt{2}$ over the analogous Method A measurement, or equivalently, it would achieve the same results but in half the total scan time. Furthermore, the single‐acquisition Method C could still be attractive for time limited acquisitions, e.g., hyperpolarized ^13^C‐MRS.

### In Vivo B_1_ Maps

We believe these are the first ^31^P‐MRS $B_{1}^{+}$‐maps made in the human heart at 7T. Therefore, there was no gold‐standard method to compare against in the heart at 7T. Instead, we validated our method in phantoms and in the leg, where it was possible to obtain reference data with a published method. In both validation experiments, the Bloch‐Siegert methods accurately matched the chosen reference methods across the whole dynamic range. The final cardiac maps present a physically reasonable range 100–1000 Hz of $\gamma B_{1}^{+}$ (5–60 μT $B_{1}^{+}$) for the experimental setup; $B_{1}^{+}$ decreases smoothly with increasing distance from the surface coil, and repeated scans in three volunteers on PCr and then on α‐ATP showed excellent reproducibility. Therefore, we deem that the implementation of a Bloch‐Siegert $B_{1}^{+}$‐mapping sequence has been successful.

### Limitations

The Fermi pulse optimization presented here is valid for the range of $B_{1}^{+}$ and SAR/ ${\left(B_{1}^{+}\right)}^{2}$ appropriate for our coil, and for T_2_ values typical in the human heart. This optimization ought to be repeated for studies that have markedly different values for these parameters. This is because there is a trade‐off between using short, high‐amplitude Fermi pulses which reduce the dead‐time and minimize T_2_‐induced signal losses but which give higher SAR and need to be placed at a greater offset frequency ω_RF_ for acceptable levels of direct excitation, and which, therefore, produce less Bloch‐Siegert phase shift for a given $B_{1}^{+}$ (and hence lead to lower $B_{1}^{+}$ precision). Or conversely, long, low‐amplitude Fermi pulses which suffer greater T_2_‐induced signal losses but produce lower SAR and may be placed at a smaller offset frequency ω_RF_ for greater $B_{1}^{+}$ precision.

The 3D‐CSI sequence localizes signals to comparatively large voxels. For a surface coil, there will be spins in each voxel that experience appreciably different $B_{1}^{+}$, and, therefore, experience different Bloch‐Siegert phase shifts. This causes intravoxel signal cancellation and, therefore, reduces the SNR efficiency of the Bloch‐Siegert method. Furthermore, because the Bloch‐Siegert phase depends on $({B_{1}^{+})}^{2}\;$, the measured voxel $B_{1}^{+}$ value will be a weighted average of $B_{1}^{+}$ within the voxel, not the mean voxel $B_{1}^{+}$ value. If a surface receive coil is used, then $B_{1}^{-}$ will also vary across each voxel. $B_{1}^{-}$ inhomogeneity will weight $B_{1}^{+}$ values measured by any method. To confirm that the additional $B_{1}^{+}$ weighting due to intravoxel difference in the Bloch‐Siegert phase shift is small, we simulated 512^3^ spin isochromats for a voxel 10 cm from the coil using the CSI protocol and RF coil described above. The Bloch‐Siegert $B_{1}^{+}$ deviated from the $B_{1}^{-}$‐weighted mean of the $B_{1}^{+}$ values in this voxel by <7%. The drop in SNR due to phase cancelation was <10% for $\gamma B_{1}^{+}<500\;\text{Hz}$ but increased to 50% at 1000 Hz. Note that this simulation is separate to the simulations described in the Theory section.

We note that during the preparation of this manuscript, a single voxel spectroscopy Bloch‐Siegert $B_{1}^{+}$ measurement technique, based on Point RESolved Spectroscopy (PRESS) has been published 31. The PRESS Bloch‐Siegert method allowed highly reproducible measurements of $B_{1}^{+}$, using the ^1^H water signal, in the hearts of six volunteers. The focus of this manuscript has been to implement these promising Bloch‐Siegert methods in a multivoxel X‐nuclear sequence, addressing the challenges $B_{1}^{+}$‐mapping in in the presence of low SNR and multiple significant peaks.

## CONCLUSIONS

We have implemented a Bloch‐Siegert spectroscopy $B_{1}^{+}$‐mapping technique, which is the first method capable of measuring per‐subject ^31^P $B_{1}^{+}$ maps in the human heart at 7T in a clinically acceptable time. We have demonstrated the optimal measurement strategy for Bloch‐Siegert spectroscopic $B_{1}^{+}$‐mapping and have numerically optimized a Fermi pulse for our application. Our method has been validated and shown to be accurate in phantoms and in skeletal muscle. It has been demonstrated successfully in the heart in a study on five normal volunteers.

## Supporting information


**Supporting Material S1.** SuppInfo_Table1FootNote_MathermaticaNotebook.nb – Original Mathematica notebook referred to in the footnote of Table 1.

**Supporting Material S2.** SuppInfo_Table1FootNote_MathermaticaNotebook.pdf – PDF rendering of SuppInfo_Table1FootNote_MathermaticaNotebook.nb

**Supporting Material S3.** SuppInfo_Table1FootNote_WolframCDF.cdf – .cdf file conversion of SuppInfo_Table1FootNote_MathermaticaNotebook.nb. This may be viewed using the free software CDF Player available from Wolfram. The software is available here: http://www.wolfram.com/cdf‐player/

**Supporting Material S4.** FermiPulseOptimisation.m – Matlab file containing the pulse optimisation process detailed in this work.

**Supporting Material S5.** Bloch_CTR_Hz.m – Auxiliary Matlab function for S4.
**Supporting Material S6.** Bloch_CTR_Hz.c – C code called by FermiPulseOptimisation.m

## References

[1] Bottomley PA . NMR spectroscopy of the human heart In: Harris RK, Wasylishen RE, editors. Encylcopedia of magnetic resonance. Chichester: John Wiley; 2009.

[2] Neubauer S . Mechanisms of disease ‐ the failing heart ‐ an engine out of fuel. N Engl J Med 2007;356:1140–1151. 17360992 10.1056/NEJMra063052

[3] Hudsmith LE , Neubauer S . Detection of myocardial disorders by magnetic resonance spectroscopy. Nat Clin Pract Card 2008;5:S49–S56. 10.1038/ncpcardio1158 18641607

[4] Neubauer S , Horn M , Cramer M , et al. Myocardial phosphocreatine‐to‐ATP ratio is a predictor of mortality in patients with dilated cardiomyopathy. Circulation 1997;96:2190–2196. 9337189 10.1161/01.cir.96.7.2190

[5] Stoll V , Clarke WT , Levelt E , Myerson SG , Robson MD , Neubauer S , Rodgers CT . 7T versus 3T phosphorus magnetic resonance spectroscopy in patients with dilated cardiomyopathy. J Cardiovasc Magn Reson 2015;17(Suppl. 1):249.

[6] Ouwerkerk R , Bottomley PA . On neglecting chemical exchange effects when correcting in vivo P‐31 MRS data for partial saturation. J Magn Reson 2001;148:425–435. 11237649 10.1006/jmre.2000.2166

[7] Bottomley PA , Ouwerkerk R , Lee RF , Weiss RG . Four‐angle saturation transfer (FAST) method for measuring creatine kinase reaction rates in vivo. Magn Reson Med 2002;47:850–863. 11979563 10.1002/mrm.10130 PMC1995126

[8] El‐Sharkawy AM , Schar M , Ouwerkerk R , Weiss RG , Bottomley PA . Quantitative cardiac P‐31 spectroscopy at 3 Tesla using adiabatic pulses. Magn Reson Med 2009;61:785–795. 19195018 10.1002/mrm.21867 PMC3084604

[9] Van De Moortele PF , Akgun C , Adriany G , Moeller S , Ritter J , Collins CM , Smith MB , Vaughan JT , Ugurbil K . B‐1 destructive interferences and spatial phase patterns at 7 T with a head transceiver array coil. Magn Reson Med 2005;54:1503–1518. 16270333 10.1002/mrm.20708

[10] Rodgers CT , Robson MD . Coil combination for receive array spectroscopy: are data‐driven methods superior to methods using computed field maps? Magn Reson Med 2016;75:473–487. 25820303 10.1002/mrm.25618 PMC4744755

[11] Chmelik M , Povazan M , Jiru F , Kukurova IJ , Dezortova M , Krssak M , Bogner W , Hajek M , Trattnig S , Valkovic L . Flip‐angle mapping of P‐31 coils by steady‐state MR spectroscopic imaging. J Magn Reson Imaging 2014;40:391–397. 24925600 10.1002/jmri.24401

[12] Spencer RGS , Fishbein KW . Measurement of spin‐lattice relaxation times and concentrations in systems with chemical exchange using the one‐pulse sequence: breakdown of the Ernst model for partial saturation in nuclear magnetic resonance spectroscopy. J Magn Reson 2000;142:120–135. 10617442 10.1006/jmre.1999.1925

[13] Smith CS , Bottomley PA , Schulman SP , Gerstenblith G , Weiss RG . Altered creatine kinase adenosine triphosphate kinetics in failing hypertrophied human myocardium. Circulation 2006;114:1151–1158. 16952984 10.1161/CIRCULATIONAHA.106.613646 PMC1808438

[14] Parasoglou P , Xia D , Chang G , Regatte RR . 3D‐mapping of phosphocreatine concentration in the human calf muscle at 7 T: comparison to 3 T. Magn Reson Med 2013;70:1619–1625. 23390003 10.1002/mrm.24616 PMC3657590

[15] Sacolick LI , Wiesinger F , Hancu I , Vogell MW . B‐1 Mapping by Bloch‐Siegert shift. Magn Reson Med 2010;63:1315–1322. 20432302 10.1002/mrm.22357 PMC2933656

[16] Emsley L , Bodenhausen G . Phase‐shifts induced by transient Bloch‐Siegert effects in NMR. Chem Phys Lett 1990;168:297–303.

[17] Bloch F , Siegert A . Magnetic resonance for nonrotating fields. Phys Rev 1940;57:522–527.

[18] Duan Q , van Gelderen P , Duyn J . Improved Bloch‐Siegert based B‐1 mapping by reducing off‐resonance shift. NMR Biomed 2013;26:1070–1078. 23355474 10.1002/nbm.2920 PMC3669656

[19] Bernstein MA , King KF , Zhou ZJ . Handbook of MRI pulse sequences. Waltham, MA: Elsevier Academic Press; 2004 1017 p.

[20] Boas ML . Mathematical methods in the physical sciences. New York: John Wiley & Sons; 1966 778 p.

[21] Rodgers CT , Clarke WT , Snyder C , Vaughan JT , Neubauer S , Robson MD . Human cardiac P‐31 magnetic resonance spectroscopy at 7 Tesla. Magn Reson Med 2014;72:304–315. 24006267 10.1002/mrm.24922 PMC4106879

[22] Cavassila S , Deval S , Huegen C , van Ormondt D , Graveron‐Demilly D . Cramer‐Rao bound expressions for parametric estimation of overlapping peaks: influence of prior knowledge. J Magn Reson 2000;143:311–320. 10729257 10.1006/jmre.1999.2002

[23] Robson MD , Tyler DJ , Neubauer S . Ultrashort TE chemical shift imaging (UTE‐CSI). Magn Reson Med 2005;53:267–274. 15678544 10.1002/mrm.20344

[24] Vanhamme L , van den Boogaart A , Van Huffel S . Improved method for accurate and efficient quantification of MRS data with use of prior knowledge. J Magn Reson 1997;129:35–43. 9405214 10.1006/jmre.1997.1244

[25] Purvis LAB , Clarke WT , Biasiolli L , Robson MD , Rodgers CT . Linewidth constraints in Matlab AMARES using per‐metabolite T_2_ and per‐voxel Delta B_0_ . In Proceedings of the 22nd Annual Meeting of ISMRM, Milan, Italy, 2014. Abstract 2885.

[26] Bogner W , Chmelik M , Schmid AI , Moser E , Trattnig S , Gruber S . Assessment of (31)P relaxation times in the human calf muscle: a comparison between 3 T and 7 T in vivo. Magn Reson Med 2009;62:574–582. 19526487 10.1002/mrm.22057

[27] Sado DM , White SK , Piechnik SK et al. Identification and assessment of Anderson‐Fabry disease by cardiovascular magnetic resonance noncontrast myocardial T1 mapping. Circ Cardiovasc Imaging 2013;6:392–398. 23564562 10.1161/CIRCIMAGING.112.000070

[28] Bottomley PA , Panjrath GS , Lai SH , Hirsch GA , Wu K , Najjar SS , Steinberg A , Gerstenblith G , Weiss RG . Metabolic rates of ATP transfer through creatine kinase (CK flux) predict clinical heart failure events and death. Sci Transl Med 2013;5:215re3. 10.1126/scitranslmed.3007328 PMC4440545 24337482

[29] Ernst RR , Anderson WA . Application of Fourier transform spectroscopy to magnetic resonance. Rev Sci Instrum 1966;37:93–102.

[30] Park DJ , Bangerter NK , Javed A , Kaggie J , Khalighi MM , Morrell GR . A statistical analysis of the Bloch‐Siegert B‐1 mapping technique. Phys Med Biol 2013;58:5673–5691. 23899515 10.1088/0031-9155/58/16/5673 PMC3845965

[31] Dokumaci AS , Pouymayou B , Kreis R , Boesch C . Motion‐insensitive determination of B1+ amplitudes based on the bloch‐siegert shift in single voxels of moving organs including the human heart. Magn Reson Med 2016;75:1867–1874. 26185908 10.1002/mrm.25763

