# Supplementary material for: Bloch‐Siegert B1+‐mapping for human cardiac 31P‐MRS at 7 Tesla
Source: Magn Reson Med. 2015 Oct 28;76(4):1047–58. doi: 10.1002/mrm.26005 (PMC5076794; doi:10.1002/mrm.26005)
Supplement: Supplementary file 2 — Supporting Material S2. SuppInfo_Table1FootNote_MathermaticaNotebook.pdf – PDF rendering of SuppInfo_Table1FootNote_MathermaticaNotebook.nb [file MRM-76-1047-s002.pdf]

Derivation of the 4 peak propagation of error equation for measurement strategy C

Form the B1 determination expression for the single-scan multi-peak method (C)

```
B1DeterminationPerPeakPair =
  Sqrt[((ϕ[a] - ϕ[b]) ) / (2 * Pi * B / 2 * Abs[(1 / ω[a] - 1 / ω[b])])]
```

$$\frac{\sqrt{\frac{\phi[a]-\phi[b]}{B}}}{\sqrt{\pi}\ \sqrt{\text{Abs}\left[\frac{1}{\omega[a]}-\frac{1}{\omega[b]}\right]}}$$

Weight this equation by the square of the difference of the inverse frequency offsets.

```
weightingTerm = (1 / ω[a] - 1 / ω[b])^2
```

$$\left(\frac{1}{\omega[a]}-\frac{1}{\omega[b]}\right)^2$$

```
weightedB1DeterminationPerPeakPair = B1DeterminationPerPeakPair * weightingTerm
```

$$\frac{\sqrt{\frac{\phi[a]-\phi[b]}{B}}\ \left(\frac{1}{\omega[a]}-\frac{1}{\omega[b]}\right)^2}{\sqrt{\pi}\ \sqrt{\text{Abs}\left[\frac{1}{\omega[a]}-\frac{1}{\omega[b]}\right]}}$$

Form the general expression as a weighted mean of each possible combination of peaks.

```
methodDGeneralB1Eqn =
  Sum[weightedB1DeterminationPerPeakPair /. {a → jDx, b → iDx},
    {iDx, 2, n}, {jDx, 1, iDx - 1}] /
   Sum[weightingTerm /. {a → mDx, b → nDx}, {nDx, 2, n}, {mDx, 1, nDx - 1}]
```

$$\frac{\sum_{iDx=2}^{n}\sum_{jDx=1}^{-1+iDx}\frac{\sqrt{\frac{-\phi[iDx]+\phi[jDx]}{B}}\ \left(-\frac{1}{\omega[iDx]}+\frac{1}{\omega[jDx]}\right)^2}{\sqrt{\pi}\ \sqrt{\text{Abs}\left[-\frac{1}{\omega[iDx]}+\frac{1}{\omega[jDx]}\right]}}}{\sum_{nDx=2}^{n}\sum_{mDx=1}^{-1+nDx}\left(\frac{1}{\omega[mDx]}-\frac{1}{\omega[nDx]}\right)^2}$$

Expand for four peaks and calculate the equation for the standard deviation of the measurement.

Expand for 4 peaks.

```
B1Eqn4Peaks = methodDGeneralB1Eqn /. n → 4
```

$$\left(\frac{\sqrt{\frac{\phi[1]-\phi[2]}{B}}\left(\frac{1}{\omega[1]}-\frac{1}{\omega[2]}\right)^2}{\sqrt{\pi}\sqrt{\mathrm{Abs}\left[\frac{1}{\omega[1]}-\frac{1}{\omega[2]}\right]}}+\frac{\sqrt{\frac{\phi[1]-\phi[3]}{B}}\left(\frac{1}{\omega[1]}-\frac{1}{\omega[3]}\right)^2}{\sqrt{\pi}\sqrt{\mathrm{Abs}\left[\frac{1}{\omega[1]}-\frac{1}{\omega[3]}\right]}}+\frac{\sqrt{\frac{\phi[2]-\phi[3]}{B}}\left(\frac{1}{\omega[2]}-\frac{1}{\omega[3]}\right)^2}{\sqrt{\pi}\sqrt{\mathrm{Abs}\left[\frac{1}{\omega[2]}-\frac{1}{\omega[3]}\right]}}+\right.$$
$$\left.\frac{\sqrt{\frac{\phi[1]-\phi[4]}{B}}\left(\frac{1}{\omega[1]}-\frac{1}{\omega[4]}\right)^2}{\sqrt{\pi}\sqrt{\mathrm{Abs}\left[\frac{1}{\omega[1]}-\frac{1}{\omega[4]}\right]}}+\frac{\sqrt{\frac{\phi[2]-\phi[4]}{B}}\left(\frac{1}{\omega[2]}-\frac{1}{\omega[4]}\right)^2}{\sqrt{\pi}\sqrt{\mathrm{Abs}\left[\frac{1}{\omega[2]}-\frac{1}{\omega[4]}\right]}}+\frac{\sqrt{\frac{\phi[3]-\phi[4]}{B}}\left(\frac{1}{\omega[3]}-\frac{1}{\omega[4]}\right)^2}{\sqrt{\pi}\sqrt{\mathrm{Abs}\left[\frac{1}{\omega[3]}-\frac{1}{\omega[4]}\right]}}\right)\Big/$$
$$\left(\left(\frac{1}{\omega[1]}-\frac{1}{\omega[2]}\right)^2+\left(\frac{1}{\omega[1]}-\frac{1}{\omega[3]}\right)^2+\left(\frac{1}{\omega[2]}-\frac{1}{\omega[3]}\right)^2+\right.$$
$$\left.\left(\frac{1}{\omega[1]}-\frac{1}{\omega[4]}\right)^2+\left(\frac{1}{\omega[2]}-\frac{1}{\omega[4]}\right)^2+\left(\frac{1}{\omega[3]}-\frac{1}{\omega[4]}\right)^2\right)$$

Apply Eq 5 (see Theory) to the previous equation. Differentiate w.r.t. each measurement variable, multiply by the uncertainty in each measurment and take the square root of the sum of the square of each term.

```
SD4PeakMethodD =
 Sqrt[(D[B1Eqn4Peaks, ϕ[1]] * Δϕ) ^2 + (D[B1Eqn4Peaks, ϕ[2]] * Δϕ) ^2 +
   (D[B1Eqn4Peaks, ϕ[3]] * Δϕ) ^2 + (D[B1Eqn4Peaks, ϕ[4]] * Δϕ) ^2]
```

$$\sqrt{\left(\left(\Delta\phi^2\left(\frac{\left(\frac{1}{\omega[1]}-\frac{1}{\omega[2]}\right)^2}{2\,B\sqrt{\pi}\sqrt{\mathrm{Abs}\left[\frac{1}{\omega[1]}-\frac{1}{\omega[2]}\right]}\sqrt{\frac{\phi[1]-\phi[2]}{B}}}+\frac{\left(\frac{1}{\omega[1]}-\frac{1}{\omega[3]}\right)^2}{2\,B\sqrt{\pi}\sqrt{\mathrm{Abs}\left[\frac{1}{\omega[1]}-\frac{1}{\omega[3]}\right]}\sqrt{\frac{\phi[1]-\phi[3]}{B}}}+\right.\right.\right.}$$
$$\left.\left.\frac{\left(\frac{1}{\omega[1]}-\frac{1}{\omega[4]}\right)^2}{2\,B\sqrt{\pi}\sqrt{\mathrm{Abs}\left[\frac{1}{\omega[1]}-\frac{1}{\omega[4]}\right]}\sqrt{\frac{\phi[1]-\phi[4]}{B}}}\right)\right)^2\Big/$$
$$\left(\left(\frac{1}{\omega[1]}-\frac{1}{\omega[2]}\right)^2+\left(\frac{1}{\omega[1]}-\frac{1}{\omega[3]}\right)^2+\left(\frac{1}{\omega[2]}-\frac{1}{\omega[3]}\right)^2+\left(\frac{1}{\omega[1]}-\frac{1}{\omega[4]}\right)^2+\right.$$
$$\left.\left(\frac{1}{\omega[2]}-\frac{1}{\omega[4]}\right)^2+\left(\frac{1}{\omega[3]}-\frac{1}{\omega[4]}\right)^2\right)^2+$$
$$\left(\Delta\phi^2\left(-\frac{\left(\frac{1}{\omega[1]}-\frac{1}{\omega[2]}\right)^2}{2\,B\sqrt{\pi}\sqrt{\mathrm{Abs}\left[\frac{1}{\omega[1]}-\frac{1}{\omega[2]}\right]}\sqrt{\frac{\phi[1]-\phi[2]}{B}}}+\frac{\left(\frac{1}{\omega[2]}-\frac{1}{\omega[3]}\right)^2}{2\,B\sqrt{\pi}\sqrt{\mathrm{Abs}\left[\frac{1}{\omega[2]}-\frac{1}{\omega[3]}\right]}\sqrt{\frac{\phi[2]-\phi[3]}{B}}}+\right.\right.$$

$$\frac{\left(\frac{1}{\omega[2]}-\frac{1}{\omega[4]}\right)^2}{2\text{ B}\sqrt{\pi}\sqrt{\text{Abs}\left[\frac{1}{\omega[2]}-\frac{1}{\omega[4]}\right]}\sqrt{\frac{\phi[2]-\phi[4]}{\text{B}}}}\Bigg)\Bigg)^2\Bigg)\Bigg/$$

$$\left(\left(\frac{1}{\omega[1]}-\frac{1}{\omega[2]}\right)^2+\left(\frac{1}{\omega[1]}-\frac{1}{\omega[3]}\right)^2+\left(\frac{1}{\omega[2]}-\frac{1}{\omega[3]}\right)^2+\left(\frac{1}{\omega[1]}-\frac{1}{\omega[4]}\right)^2+\right.$$

$$\left.\left(\frac{1}{\omega[2]}-\frac{1}{\omega[4]}\right)^2+\left(\frac{1}{\omega[3]}-\frac{1}{\omega[4]}\right)^2\right)^2+$$

$$\Bigg(\Delta\phi^2\Bigg(-\frac{\left(\frac{1}{\omega[1]}-\frac{1}{\omega[4]}\right)^2}{2\text{ B}\sqrt{\pi}\sqrt{\text{Abs}\left[\frac{1}{\omega[1]}-\frac{1}{\omega[4]}\right]}\sqrt{\frac{\phi[1]-\phi[4]}{\text{B}}}}-\frac{\left(\frac{1}{\omega[2]}-\frac{1}{\omega[4]}\right)^2}{2\text{ B}\sqrt{\pi}\sqrt{\text{Abs}\left[\frac{1}{\omega[2]}-\frac{1}{\omega[4]}\right]}\sqrt{\frac{\phi[2]-\phi[4]}{\text{B}}}}-$$

$$\frac{\left(\frac{1}{\omega[3]}-\frac{1}{\omega[4]}\right)^2}{2\text{ B}\sqrt{\pi}\sqrt{\text{Abs}\left[\frac{1}{\omega[3]}-\frac{1}{\omega[4]}\right]}\sqrt{\frac{\phi[3]-\phi[4]}{\text{B}}}}\Bigg)^2\Bigg)\Bigg/$$

$$\left(\left(\frac{1}{\omega[1]}-\frac{1}{\omega[2]}\right)^2+\left(\frac{1}{\omega[1]}-\frac{1}{\omega[3]}\right)^2+\left(\frac{1}{\omega[2]}-\frac{1}{\omega[3]}\right)^2+\left(\frac{1}{\omega[1]}-\frac{1}{\omega[4]}\right)^2+\right.$$

$$\left.\left(\frac{1}{\omega[2]}-\frac{1}{\omega[4]}\right)^2+\left(\frac{1}{\omega[3]}-\frac{1}{\omega[4]}\right)^2\right)^2+$$

$$\Bigg(\Delta\phi^2\Bigg(-\frac{\left(\frac{1}{\omega[1]}-\frac{1}{\omega[3]}\right)^2}{2\text{ B}\sqrt{\pi}\sqrt{\text{Abs}\left[\frac{1}{\omega[1]}-\frac{1}{\omega[3]}\right]}\sqrt{\frac{\phi[1]-\phi[3]}{\text{B}}}}-\frac{\left(\frac{1}{\omega[2]}-\frac{1}{\omega[3]}\right)^2}{2\text{ B}\sqrt{\pi}\sqrt{\text{Abs}\left[\frac{1}{\omega[2]}-\frac{1}{\omega[3]}\right]}\sqrt{\frac{\phi[2]-\phi[3]}{\text{B}}}}+$$

$$\frac{\left(\frac{1}{\omega[3]}-\frac{1}{\omega[4]}\right)^2}{2\text{ B}\sqrt{\pi}\sqrt{\text{Abs}\left[\frac{1}{\omega[3]}-\frac{1}{\omega[4]}\right]}\sqrt{\frac{\phi[3]-\phi[4]}{\text{B}}}}\Bigg)^2\Bigg)\Bigg/$$

$$\left(\left(\frac{1}{\omega[1]}-\frac{1}{\omega[2]}\right)^2+\left(\frac{1}{\omega[1]}-\frac{1}{\omega[3]}\right)^2+\left(\frac{1}{\omega[2]}-\frac{1}{\omega[3]}\right)^2+\left(\frac{1}{\omega[1]}-\frac{1}{\omega[4]}\right)^2+\right.$$

$$\left.\left(\frac{1}{\omega[2]}-\frac{1}{\omega[4]}\right)^2+\left(\frac{1}{\omega[3]}-\frac{1}{\omega[4]}\right)^2\right)^2\Bigg)$$
